# Diverse geroprotectors differently affect a mechanism linking cellular aging to cellular quiescence in budding yeast

**DOI:** 10.18632/oncotarget.28256

**Published:** 2022-07-28

**Authors:** Anna Leonov, Rachel Feldman, Amanda Piano, Anthony Arlia-Ciommo, Jennifer Anne Baratang Junio, Emmanuel Orfanos, Tala Tafakori, Vicky Lutchman, Karamat Mohammad, Sarah Elsaser, Sandra Orfali, Harshvardhan Rajen, Vladimir I. Titorenko

**Affiliations:** ^1^Department of Biology, Concordia University, Montreal, Quebec H4B 1R6, Canada

**Keywords:** cellular aging, cellular quiescence, longevity, gerotargets, geroprotectors

## Abstract

We propose a hypothesis of a mechanism linking cellular aging to cellular quiescence in chronologically aging budding yeast. Our hypothesis posits that this mechanism integrates four different processes, all of which are initiated after yeast cells cultured in a medium initially containing glucose consume it. Quiescent cells that develop in these cultures can be separated into the high- and low-density sub-populations of different buoyant densities. Process 1 of the proposed mechanism consists of a cell-cycle arrest in the G_1_ phase and leads to the formation of high-density quiescent cells. Process 2 results in converting high-density quiescent cells into low-density quiescent cells. Processes 3 and 4 cause a fast or slow decline in the quiescence of low- or high-density quiescent cells, respectively. Here, we tested our hypothesis by assessing how four different geroprotectors influence the four processes that could link cellular aging to cellular quiescence. We found that these geroprotectors differently affect processes 1 and 2 and decelerate processes 3 and 4. We also found that a rise in trehalose within quiescent yeast contributes to chronological aging and quiescence maintenance. These data collectively provide conclusive evidence for a mechanistic link between cellular aging and cellular quiescence.

## INTRODUCTION

Specific cell-extrinsic and cell-intrinsic anti-mitogenic factors can cause a temporary cell cycle arrest in unicellular and multicellular eukaryotes [1–10]. These cells then enter a reversible G_0_ state of quiescence [1–10].

Quiescent adult stem cells in metazoans are long-lived because of their resistance to various stresses and toxicities [2, 6, 7, 11–18]. These cells also retain the ability to re-enter the cell cycle and resume proliferation in response to specific pro-mitogenic stimuli [2, 3, 7, 11, 12, 16, 19–29]. Such infrequent proliferation of quiescent adult stem cells allows them to self-renew while maintaining the state of quiescence [2, 3, 7, 11, 12, 16, 19–29]. The infrequent proliferation of quiescent adult stem cells often also yields actively dividing daughter progenitor cells that can undergo a terminal differentiation [2, 3, 7, 11, 12, 16, 19–29].

The maintenance of an appropriate number of quiescent adult stem cells that can sustain the state of quiescence, resist stresses, self-renew and form progenitor cells for terminal differentiation contributes to the growth, development, tissue repair and regeneration, and longevity assurance in metazoans [2, 4, 7, 21, 22, 26, 29–34]. An aging-associated numerical and functional decline of quiescent adult stem cells is an essential contributor to the pathophysiology of many diseases of old age in mammals and humans [7, 11, 13, 14, 21, 30–32, 34–55]. The genetic, dietary, and pharmacological interventions that can slow such decline can also slow cellular and organismal aging and delay the onset of aging-associated diseases [7, 11, 13, 14, 21, 30–32, 34–55].

All microorganisms (including yeasts) in natural environments outside of the laboratory can undergo a reversible transition between the states of cellular quiescence and proliferation in response to the variations in nutrient availability and other environmental cues [1, 3, 56–64]. The yeast *Saccharomyces cerevisiae*, a unicellular eukaryote amenable to genetic and biochemical analyses, is a valuable model organism for elucidating mechanisms of cellular quiescence under laboratory conditions [1, 3, 60, 62, 65, 66].

Mechanisms of cellular quiescence in *S. cerevisiae* have been traditionally studied in yeast cells that are not limited in calorie supply. Under these so-called non-caloric restriction (non-CR) conditions, *S. cerevisiae* cells are aerobically cultured in a nutrient-rich liquid medium initially containing 2% glucose [67–70]. After consuming glucose as a carbon source, these yeast cells slow their growth and enter a diauxic shift period [68, 71]. Some yeast cells in the culture that enters the diauxic shift undergo a cell-cycle arrest at the checkpoint “START A” in late G_1,_ and the culture becomes differentiated into the sub-populations of quiescent (Q) and non-quiescent (NQ) cells [62, 72–74]. The properties of Q and NQ cell sub-populations formed in yeast cultures under non-CR conditions are different. These cell sub-populations differ from each other in size, morphology, density, metabolism, transcriptional pattern, stress resistance, proteostatic control, cytoskeleton arrangement, mitochondrial morphology, signal transduction design, and susceptibility to the apoptotic form of regulated cell death (RCD) [5, 62, 64, 72–91]. An intricate signaling network coordinates the diversification of properties that distinguish the Q and NQ cell sub-populations formed in yeast cultures not limited in calorie supply [1, 82, 92–108]. A body of evidence indicates that the entry into and advancement through a quiescence program taking place in yeast cells under non-CR conditions are essential contributors to their chronological aging [1, 62, 72–75, 80, 82, 85, 88–90, 93, 100, 102, 103, 105, 108].

We have introduced a new yeast model for studying mechanisms linking cellular aging to cellular quiescence [109, 110]. In the new model, these potentially existing mechanisms are studied in yeast cells limited in calorie supply because they are cultured in a nutrient-rich medium initially containing 0.2% glucose. CR is known to delay chronological aging and prolong the longevity of *S. cerevisiae* [67, 69, 70, 111–113]. This low-calorie diet is a robust geroprotective intervention that extends organisms’ lifespan and health span across phyla [111, 114–123].

**Figure 1:**
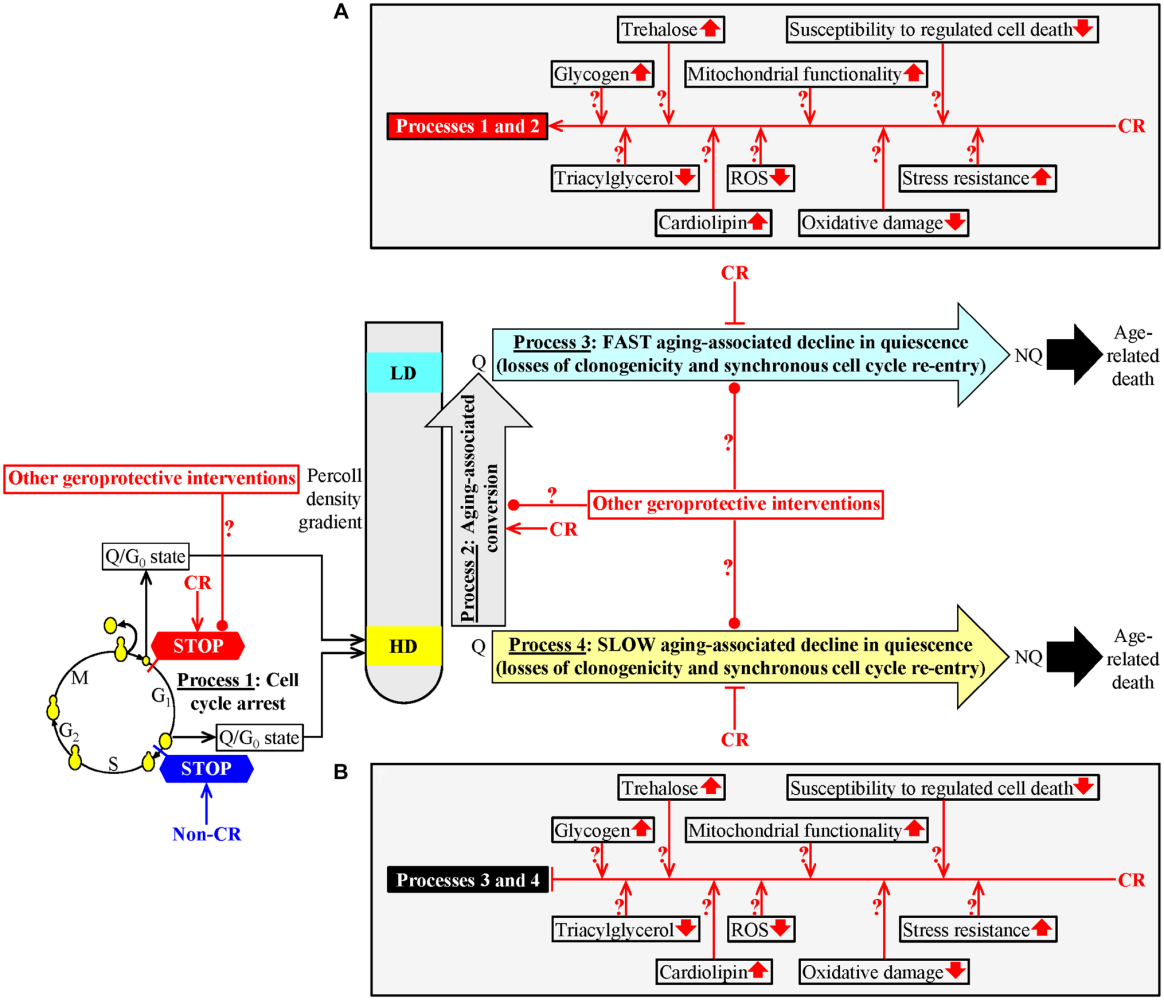
A hypothetical model for the four processes linking cellular aging to cellular quiescence. We hypothesized that these processes converge into a mechanism that links cellular aging to cellular quiescence in chronologically aging budding yeast. To test our hypothesis, we had the following two objectives. The first objective of this study was to investigate how geroprotective interventions other than caloric restriction (CR) affect each of these processes. The second objective of this study was to test how some CR-specific biochemical traits of quiescent (Q) cells contribute to the abilities of CR and LCA to slow down a decline in the cellular quiescence of chronologically aging budding yeast. Process 1: CR creates a sub-population of high-density Q cells by arresting the cell-division cycle of some cells and eliciting their entry into the G_0_ state in early G_1_; yet, the development of such a cell sub-population under non-CR conditions happens by a cell-cycle arrest in late G_1_. Process 2: CR accelerates the conversion of high-density Q cells into low-density Q cells. CR postpones a fast transformation of low-density Q cells into low-density NQ cells (process 3) and a slow conversion of high-density Q cells into high-density NQ cells (process 4). See the text for more details. The metabolic traits of Q cells that can contribute to the different effects of CR and LCA on processes 1 and 2 (**A**) and their similar effects on processes 3 and 4 (**B**) are shown. Other Abbreviations: HD: high-density cells; LD: low-density cells. ↑an increase ↓a decrease →→ activation arrows ˧˧inhibition bars.

We used density gradient centrifugation to purify Q and NQ cells from *S. cerevisiae* cultured under CR or non-CR conditions [109]. We recovered these cells on different days of the chronological aging process [109]. We found that the CR diet regulates the following four processes that could link cellular aging to cellular quiescence (Figure 1). First, CR creates a stem cell niche of high-density Q cells, arrests their cell cycle and causes their entry into the G_0_ state at a checkpoint in early G_1_ (Figure 1, process 1) [109]. In contrast, the formation of such a stem cell niche of high-density Q cells under non-CR conditions occurs by a cell-cycle arrest in late G_1_ [1, 109]. Second, high-density Q cells undergo conversion into low-density Q cells as early as in the logarithmic (L) growth phase of a yeast culture under CR conditions (Figure 1, process 2) [109]. Yet, such conversion occurs only in the stationary (ST) phase of a yeast culture that is not limited in calorie supply [109]. Third, CR slows the conversion of low-density Q cells into low-density NQ cells (Figure 1, process 3) [109]. This aging-associated process 3 is fast [109]. Fourth, CR decelerates the transformation of high-density Q cells into high-density NQ cells (Figure 1, process 4) [109]. This aging-associated process is slow [109]. An aging-associated decline in quiescence during processes 3 and 4 was assessed by monitoring two fundamental characteristics of Q cells that distinguish them from NQ cells. These characteristics include the following: 1) the clonogenicity (*i.e.*, the ability of a cell to form a colony after being transferred from a nutrient-depleted liquid medium to a surface of a nutrient-rich solid medium) and 2) synchronous cell cycle re-entry (*i.e.*, the ability of a cell population to synchronously re-enter the mitotic cell cycle after being transferred from a nutrient-depleted liquid medium to a nutrient-rich liquid medium) (Figure 1, processes 3 and 4) [109]. The CR-dependent delays of the aging-associated processes 3 and 4 coincide with changes in several key traits of low- and high-density Q cells caused by CR [109]. These CR-driven traits of Q cells include a rise in glycogen and trehalose concentrations, a decline in the concentrations of triacylglycerols (TAG), an increase in cardiolipin (CL) concentrations, a decrease in the concentrations of reactive oxygen species (ROS), an improvement of mitochondrial functionality, reduced oxidative damage to macromolecules, a rise in cell resistance to long-term thermal and oxidative stresses, and a decline in cell susceptibility to apoptotic and liponecrotic forms of regulated cell death (Figure 1A and 1B) [109].

Based on the above findings, we proposed a hypothesis that processes 1, 2, 3 and 4 converge into a mechanism that links cellular aging to cellular quiescence in chronologically aging budding yeast (Figure 1) [110]. According to our hypothesis, CR delays yeast chronological aging in part because it targets this hypothetical mechanism operating within Q cells (Figure 1) [110]. Our hypothesis also posits that the aging-delaying effects of geroprotective interventions other than CR are partly due to their ability to regulate the four different processes integrated into the mechanism linking cellular aging to cellular quiescence. Our hypothesis further suggests that the aging-delaying effect of CR (and, perhaps, of other geroprotective interventions) is caused by its ability to alter the specific traits characteristic of Q cells, thereby changing the efficiencies of processes 1, 2, 3 and/or 4 (Figure 1A and 1B) [110].

The present study had the following two objectives aimed at testing our hypothesis.

Our first objective was to assess how geroprotective interventions other than CR influence processes 1, 2, 3 and 4 that could link cellular aging to cellular quiescence (Figure 1). To attain this objective, we examined the effects of lithocholic acid (LCA) and the single-gene deletion mutations *tor1Δ* and *ras2Δ* on these processes. Each of these geroprotectors delays chronological aging and extends the longevity of *S. cerevisiae* [124–126]. We tested each of them in yeast cultured under non-CR conditions on 2% glucose, under which all these interventions exhibit strong geroprotective effects [124–126].

In experiments pursuing our first objective, we found that CR and the *ras2Δ* mutation (if present in yeast cultured under non-CR conditions) influence processes 1 and 2 potentially linking cellular aging to cellular quiescence differently than LCA and the *tor1Δ* mutation (if added to or present in yeast cultured under non-CR conditions, respectively). Yet, our experiments following the first objective also revealed that each of the four tested geroprotective interventions (*i.e.*, CR, LCA, *tor1Δ* and *ras2Δ*) has a similar effect on processes 3 and 4 possibly linking cellular aging to cellular quiescence. Based on these findings, we thought that there are two ways of slowing down yeast chronological aging by the four tested geroprotectors; one way is specific for CR and the *ras2Δ* mutation, whereas the other way is characteristic for LCA and the *tor1Δ* mutation. Therefore, we selected CR and LCA as the aging-delaying interventions to further investigate the two ways through which diverse geroprotectors postpone yeast chronological aging by differently targeting the mechanism that could link cellular aging to cellular quiescence.

Our second objective was to test a hypothesis that specific metabolic traits of Q cells can contribute to the different effects of CR and LCA on processes 1 and 2 and their similar effects on processes 3 and 4 (Figure 1A and 1B).

To make a first step toward attaining this objective, we assessed the contributory roles of two CR-specific changes in metabolic traits of Q cells. These traits included the increased intracellular concentrations of glycogen and trehalose within Q cells. We hypothesized that the abilities of CR and LCA to regulate the four processes of a cellular quiescence program are responsible for their abilities to slow yeast chronological aging. Therefore, we assessed the contributions of the increased intracellular concentrations of glycogen and trehalose within Q cells to the CR- and LCA-driven changes in cellular quiescence and to the CR- and LCA-promoted slowdowns of yeast chronological aging. To perform this assessment, we tested the effects of the single-gene deletion mutations differently affecting intracellular glycogen and trehalose on yeast quiescence and longevity.

## RESULTS

### CR and the *ras2Δ* mutation influence the formation of high-density Q cells differently than LCA and the *tor1Δ* mutation

Process 1 linking cellular aging to cellular quiescence could lead to the formation of a stem cell niche of high-density Q cells (Figure 1) [109]. The sub-populations of high-density Q cells contain unbudded cells, especially the sub-populations recovered from yeast cultures that pass the L growth phase [109]. Process 1 consists of a cell-cycle arrest at a checkpoint in the G_1_ phase [1, 109, 127]. In yeast cultured under non-CR conditions on 2% glucose, process 1 occurs at the checkpoint “START A” in late G_1_ and results in the formation of large-sized (cell diameter ~ 5–6 μm) high-density Q cells [1, 109, 127]. In yeast cultured under CR conditions on 0.2% glucose, process 1 occurs at a different checkpoint in the early G_1_ phase. Judging from the small size (cell diameter ~ 3–3.5 μm) of high-density Q cells observed in CR yeast cultures, CR arrests the cell cycle and causes entry into the G_0_ state of quiescence at a checkpoint in early G_1_ [109].

We sought to investigate how geroprotective interventions other than CR influence process 1. These additional geroprotective interventions included LCA and the single-gene deletion mutations *tor1Δ* and *ras2Δ*. Each of them was assessed in yeast cultured under non-CR conditions on 2% glucose. Of note, all these interventions exhibit strong geroprotective effects under non-CR conditions of culturing [124–126]. We used centrifugation in the Percoll density gradient to purify the sub-populations of high-density Q cells from yeast cultures of different chronological ages. These cell sub-populations were subjected to differential interference contrast (DIC) microscopical examination and subsequent morphometric analysis.

**Figure 2:**
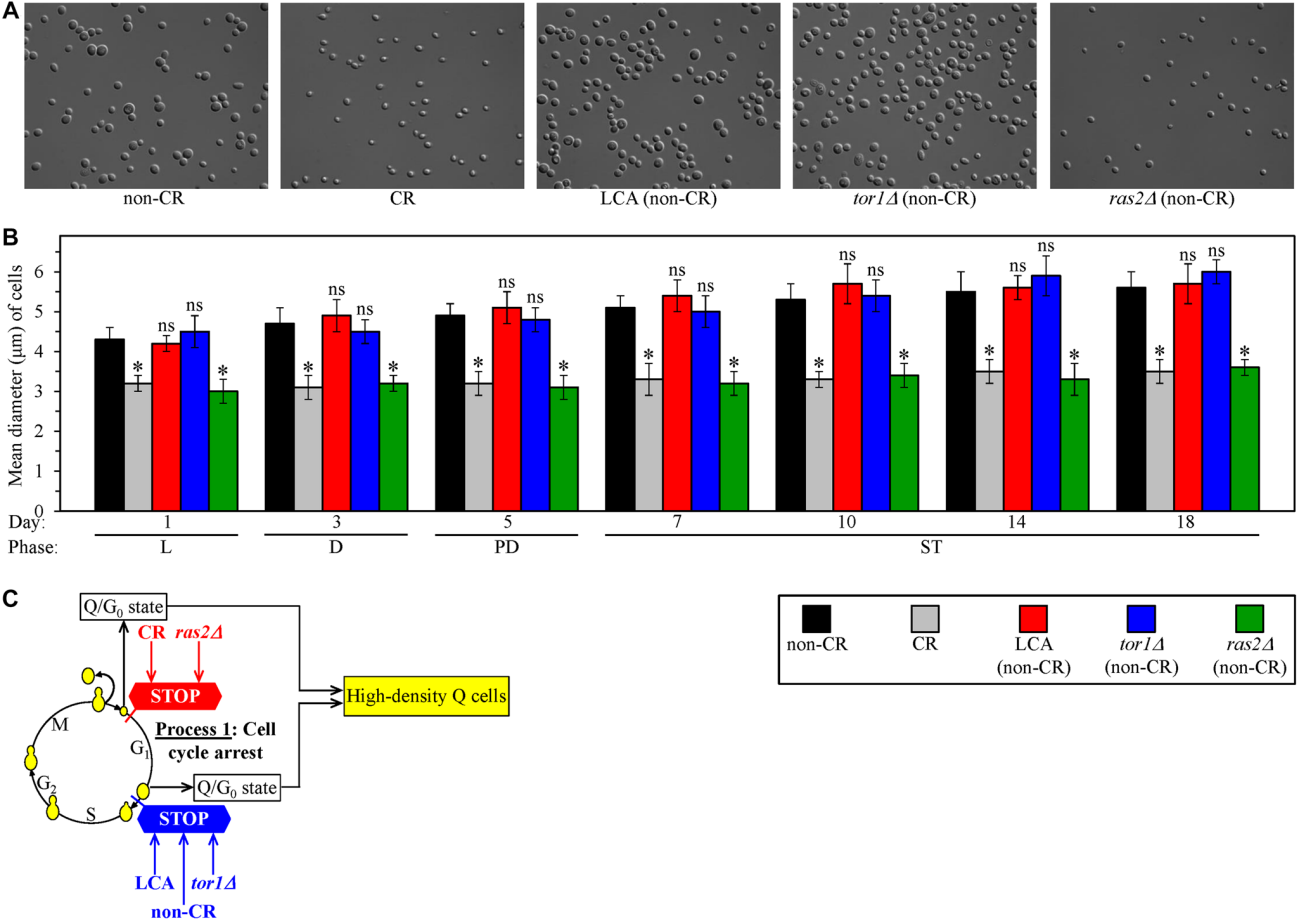
Caloric restriction (CR) and the *ras2Δ* mutation under non-CR conditions cause the formation of high-density Q cells by arresting the cell cycle at a checkpoint in early G_1_. In contrast, lithocholic acid (LCA) and the *tor1Δ* mutation elicit the formation of high-density Q cells under non-CR conditions by arresting the cell cycle at a checkpoint in late G_1_. Wild-type (WT) yeast cells were cultured in the nutrient-rich YP medium initially containing 0.2% glucose (CR conditions) or 2% glucose (non-CR conditions) with or without LCA. The *tor1Δ* and *ras2Δ* mutant cells were cultured in the nutrient-rich YP medium initially containing 2% glucose (non-CR conditions) without LCA. Culture aliquots were recovered from the logarithmic (L), diauxic (D), post-diauxic (PD) or stationary (ST) growth phase. High-density Q cells were purified from these culture aliquots using centrifugation in Percoll density gradient, as described in Materials and Methods. Differential interference contrast micrographs of high-density Q cells recovered from the ST growth phase on day 7 (**A**) and mean diameters of high-density Q cells recovered from different growth phases (**B**) are shown. (**C**) A model for how CR, LCA (under non-CR conditions), *tor1Δ* (under non-CR conditions) and *ras2Δ* (under non-CR conditions) cause the formation of high-density Q cells by arresting the cell cycle at specific checkpoints in early (red) or late (blue) G_1_. Data in B are presented as means ± SEM (*n* = 3; ^*^ *p* < 0.05; ns, not significant).

We found that high-density Q cells in CR cultures of wild-type (WT) strain and in non-CR cultures of the untreated *ras2Δ* mutant strain remain significantly smaller than high-density Q cells in three other cultures tested on any day of cell recovery (Figure 2A and 2B). These three other cultures were the non-CR culture of WT stain without LCA, the non-CR culture of WT stain with LCA and the non-CR culture of the untreated *tor1Δ* mutant strain (Figure 2A and 2B).

Judging from the observed differences in size between high-density Q cells formed in tested cultures, we concluded that the formation of small Q cells in non-CR cultures of the untreated *ras2Δ* mutant strain is due to the cell-cycle arrest at a checkpoint in early G_1_. Because high-density Q cells formed in the CR culture of WT strain have a similar small size, it is conceivable that CR and *ras2Δ* arrest the cell cycle and cause the entry into quiescence at the same checkpoint in early G_1_ (Figure 2C). Furthermore, the formation of high-density Q cells in non-CR cultures of WT stain treated with LCA and the untreated *tor1Δ* mutant strain was likely due to the cell-cycle arrest at a checkpoint in late G_1_. Because high-density Q cells formed in the non-CR culture of untreated WT strain have a similar large size, it is plausible that non-CR conditions, LCA and *tor1Δ* arrest the cell cycle and elicit the entry into quiescence at the same checkpoint in late G_1_ (Figure 2C).

### CR and the *ras2Δ* mutation affect the conversion of high-density Q cells into low-density Q cells in a different way than LCA and the *tor1Δ* mutation

Centrifugation in the Percoll density gradient can separate high-density Q cells from low-density Q cells because these two cell sub-populations have different buoyant densities [109]. Process 2 linking cellular aging to cellular quiescence could result in an age-related conversion of high-density Q cells into low-density Q cells (Figure 1) [109]. We previously found that the percentage of high-density Q cells declines, and the percentage of low-density Q cells rises during chronological aging of yeast cultured under non-CR conditions on 2% glucose [109]. We also found that the age-related conversion of high-density Q cells into low-density Q cells in process 2 is accelerated in yeast cultured under CR conditions on 0.2% glucose [109].

We wanted to explore how LCA and the single-gene deletion mutations *tor1Δ* and *ras2Δ* affect process 2. As we mentioned above, each of these three geroprotective interventions was tested under non-CR conditions of culturing. Under such conditions, each o them significantly slows down chronological aging in *S. cerevisiae* [124–126]. We cultured WT yeast cells under CR conditions on 0.2% glucose, under non-CR conditions on 2% glucose, and under non-CR conditions on 2% glucose with LCA. We also cultured the *tor1Δ* and *ras2Δ* mutant cells under non-CR conditions on 2% glucose. Using centrifugation in the Percoll density gradient, we purified the sub-populations of high-density Q cells and low-density Q cells from the above yeast cultures of different chronological ages. We then used a cell counter to determine the percentage of high-density Q cells in these differently aged cell sub-populations.

**Figure 3:**
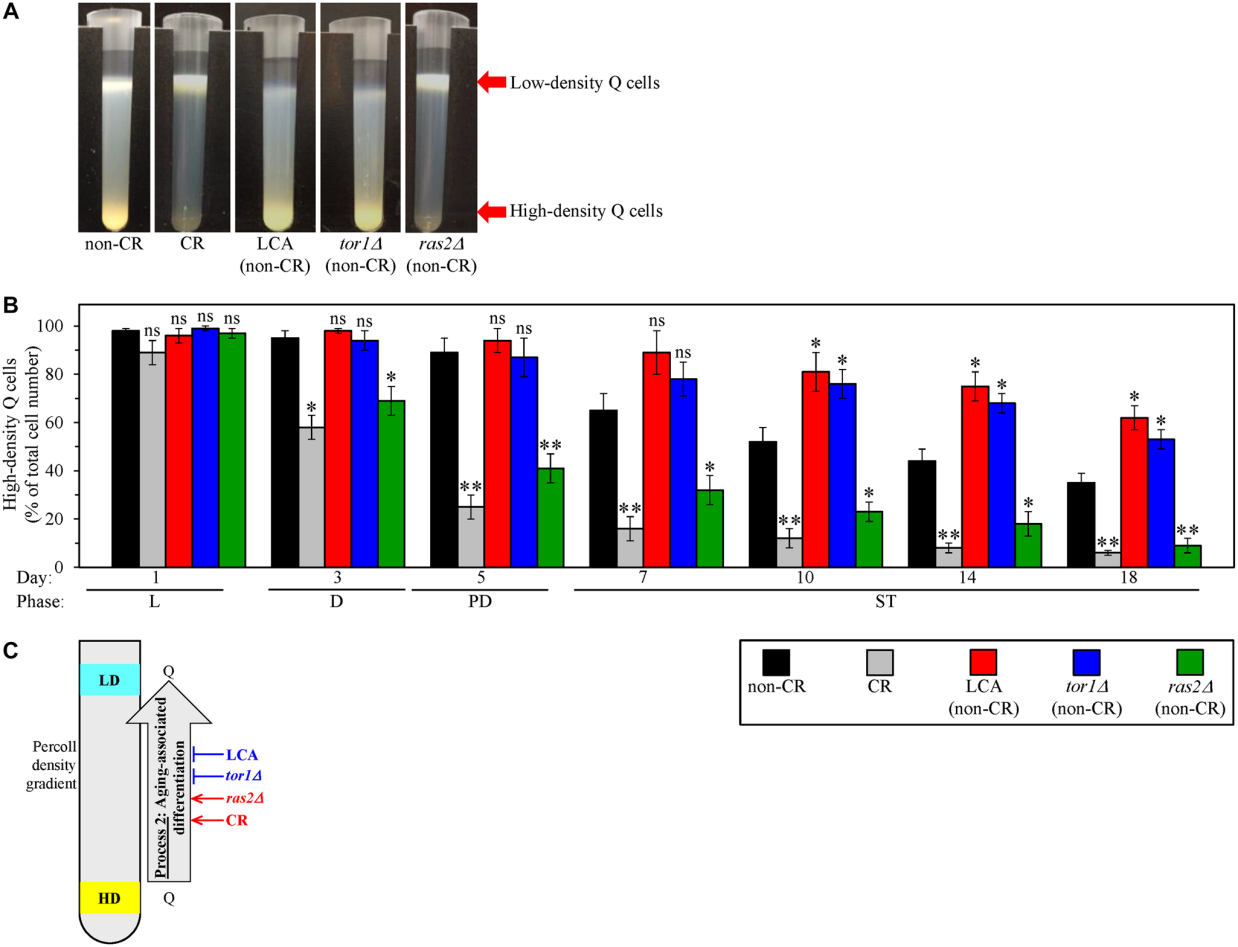
Caloric restriction (CR) and the *ras2Δ* mutation under non-CR conditions accelerate the process 2 of an age-related conversion of high-density quiescent (Q) cells into low-density Q cells. In contrast, lithocholic acid (LCA) and the *tor1Δ* mutation slow down process 2 under non-CR conditions. Wild-type (WT) yeast cells were cultured in the nutrient-rich YP medium initially containing 0.2% glucose (CR conditions) or 2% glucose (non-CR conditions) with or without LCA. The *tor1Δ* and *ras2Δ* mutant cells were cultured in the nutrient-rich YP medium initially containing 2% glucose (non-CR conditions) without LCA. Aliquots of differently aged cell populations were recovered from the logarithmic (L), diauxic (D), post-diauxic (PD) or stationary (ST) growth phase. High-density Q cells and low-density Q cells were purified from these cell populations with the help of the centrifugation in Percoll density gradient, as described in Materials and Methods. A cell counter was used to determine the percentage of high-density Q cells in each of these differently aged populations. (**A**) Percoll density gradients used to purify high-density Q cells and low-density Q cells from WT or mutant cell populations recovered on day 10 (the ST growth phase) of culturing are shown. (**B**) The percentage of high-density Q cells in differently aged WT or mutant cell populations. (**C**) A model for how CR, LCA (under non-CR conditions), *tor1Δ* (under non-CR conditions) and *ras2Δ* (under non-CR conditions) affect the process 2 of an age-related conversion of high-density Q cells into low-density Q cells. Data in B are presented as means ± SEM (*n* = 3; ^*^ *p* < 0.05; ns, not significant). Other Abbreviations: HD: high-density cells; LD: low-density cells.

We found that all five tested cultures contain the sub-populations of high-density Q and low-density Q cells during the logarithmic (L), diauxic (D), post-diauxic (PD) and stationary (ST) growth phases, *i.e.*, through the entire chronological lifespan (CLS) (Figure 3A and 3B). Furthermore, the percentage of high-density Q cells declined with chronological age in all these cultures (Figure 3B). Moreover, since day 3 (the D growth phase) of culturing, the percentage of high-density Q cells in the untreated cultures of WT yeast under CR conditions and of *ras2Δ* mutant yeast under non-CR conditions was significantly lower than that in the control culture of untreated WT yeast under non-CR conditions (Figure 3B). We also found that, since day 10 (the ST phase) of culturing, the percentage of high-density Q cells in the cultures of WT yeast under non-CR conditions with LCA and of untreated *tor1Δ* mutant yeast under non-CR conditions exceeds that in the control culture of untreated WT yeast under non-CR conditions (Figure 3B).

Collectively, these findings indicate that CR and *ras2Δ* (under non-CR conditions) accelerate the process 2 of an age-related conversion of high-density Q cells into low-density Q cells (Figure 3C). In contrast, LCA and *tor1Δ* (both under non-CR conditions) slow down process 2 (Figure 3C).

### All four tested geroprotectors decelerate the aging-associated processes of a fast deterioration in the quiescence of low-density Q cells and a slow decline in the quiescence of high-density Q cells

Process 3 linking cellular aging to cellular quiescence could lead to a fast aging-associated deterioration in the quiescence of low-density Q cells (Figure 1) [109]. Process 4 linking cellular aging to cellular quiescence could cause a slow aging-associated decline in the quiescence of high-density Q cells (Figure 1) [109]. We previously found that processes 3 and 4 are slowed down in yeast cultured under CR conditions on 0.2% glucose [109].

We sought to investigate how LCA and the single-gene deletion mutations *tor1Δ* and *ras2Δ* influence processes 3 and 4 under non-CR conditions of culturing.

We first compared the effects of CR, LCA, *tor1Δ* and *ras2Δ* on an aging-associated decline in the clonogenicity of low- and high-density Q cells. Clonogenicity is a hallmark of quiescence. Q cells are clonogenic because, unlike NQ cells, they can form a colony after being transferred from a nutrient-depleted liquid medium to a surface of a nutrient-rich solid medium [62, 109].

We cultured WT and mutant yeast strains under the conditions described in the previous section and collected culture aliquots of different chronological ages. We used centrifugation in the Percoll density gradient to purify the sub-populations of high- and low-density Q cells from these aliquots. We then assessed the clonogenicities of these cell sub-populations with the help of a plating assay for reproductive (colony-forming) capability.

**Figure 4:**
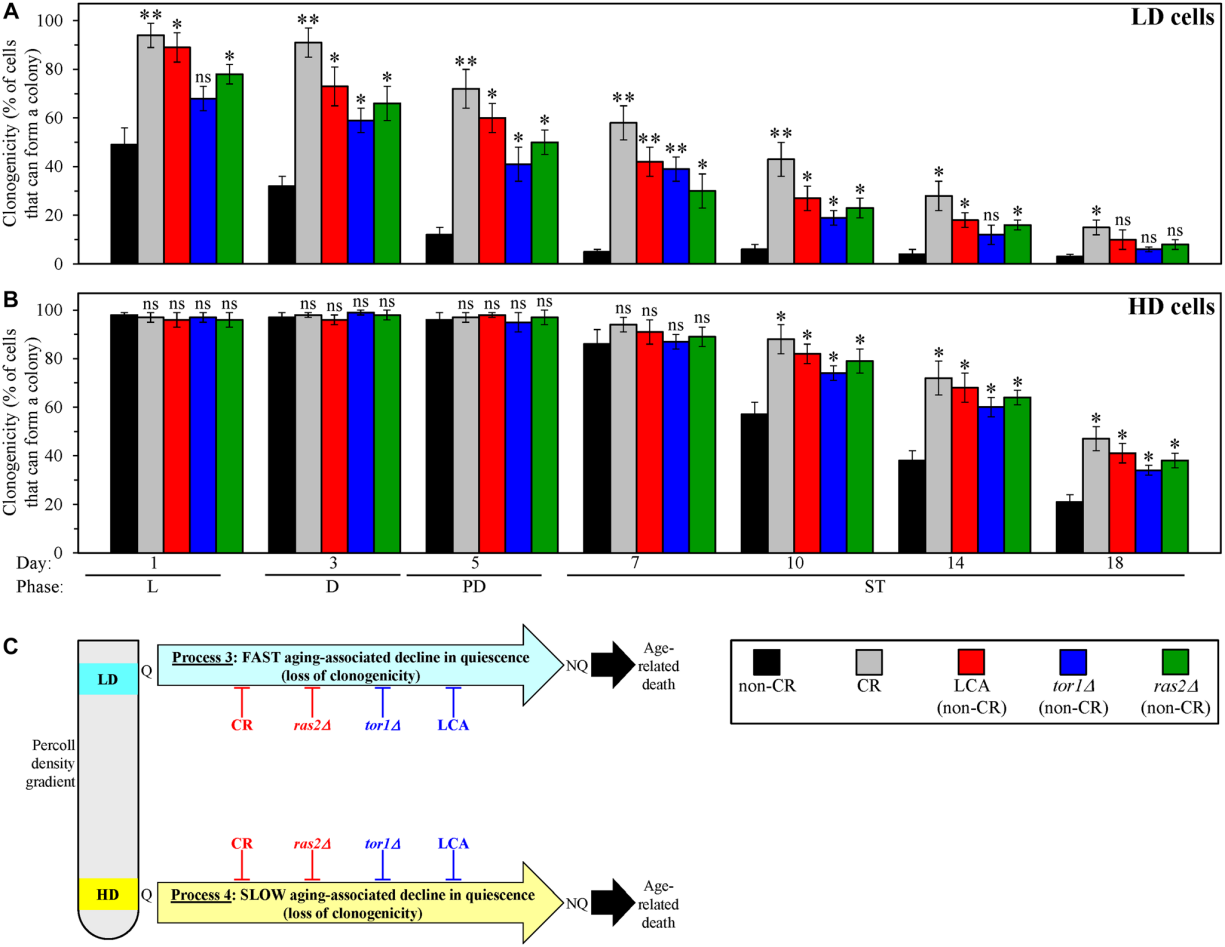
Caloric restriction (CR), lithocholic acid (LCA), the *tor1Δ* mutation and the *ras2Δ* mutation postpone an aging-associated decline in the clonogenicities of low-density quiescent (Q) cells and high-density Q cells during processes 3 and 4, respectively. Wild-type (WT) yeast cells were cultured in the nutrient-rich YP medium initially containing 0.2% glucose (CR conditions) or 2% glucose (non-CR conditions) with or without LCA. The *tor1Δ* and *ras2Δ* mutant cells were cultured in the nutrient-rich YP medium initially containing 2% glucose (non-CR conditions) without LCA. Aliquots of differently aged cell populations were recovered from the logarithmic (L), diauxic (D), post-diauxic (PD) or stationary (ST) growth phase. High-density Q cells and low-density Q cells were purified from these cell populations with the help of the centrifugation in Percoll density gradient, as described in Materials and Methods. A plating assay for reproductive (colony forming) capability, which is described in Materials and Methods, was used to measure the clonogenicities of low-density Q cells (**A**) and high-density Q cells (**B**) in differently aged WT or mutant cell populations. (**C**) A model for how CR, LCA (under non-CR conditions), *tor1Δ* (under non-CR conditions) and *ras2Δ* (under non-CR conditions) affect an aging-associated deterioration in the clonogenicities of low-density Q cells and high-density Q cells during processes 3 and 4, respectively. Data in A and B are presented as means ± SEM (*n* = 3; ^*^ *p* < 0.05; ^**^ *p* < 0.01; ns, not significant). Other Abbreviations: HD: high-density cells; LD: low-density cells; NQ: non-quiescent cells.

We found that, through the entire chronological lifespan, the CR, LCA, *tor1Δ* and *ras2Δ* geroprotectors slow down the aging-associated process 3 of a fast deterioration in the clonogenicity (*i.e.*, quiescence) of low-density Q cells (Figure 4A). We also found that, since day 10 (the ST growth phase) of culturing, all these geroprotectors decelerate the aging-associated process 4 of a slow decline in the clonogenicity (*i.e.*, quiescence) of high-density Q cells (Figure 4B).

Based on these observations, we concluded that CR, LCA, *tor1Δ* and *ras2Δ* slow down an aging-associated deterioration in the clonogenicities of low-density Q cells and high-density Q cells during processes 3 and 4, respectively (Figure 4C).

We also examined how CR, LCA, *tor1Δ* and *ras2Δ* influence an aging-associated decline in the abilities of low- and high-density Q cells to synchronously re-enter the mitotic cell cycle after being transferred from a nutrient-depleted liquid medium to a nutrient-rich liquid medium. The synchronous cell cycle re-entry is another hallmark of quiescence [62, 109]. This essential trait of Q cells distinguishes them from NQ cells [62, 109].

WT and mutant yeast strains were cultured under the conditions described in the previous section. Culture aliquots of diverse chronological ages were collected on different days of culturing. The sub-populations of high- and low-density Q cells were purified from these aliquots with the help of Percoll density gradient centrifugation.

We then assessed the effects of CR, LCA, *tor1Δ* and *ras2Δ* on the abilities of purified high- and low-density Q cells to synchronously re-enter the mitotic cell cycle after cell transfer into a fresh medium and incubation for 1 to 4 h.

**Figure 5:**
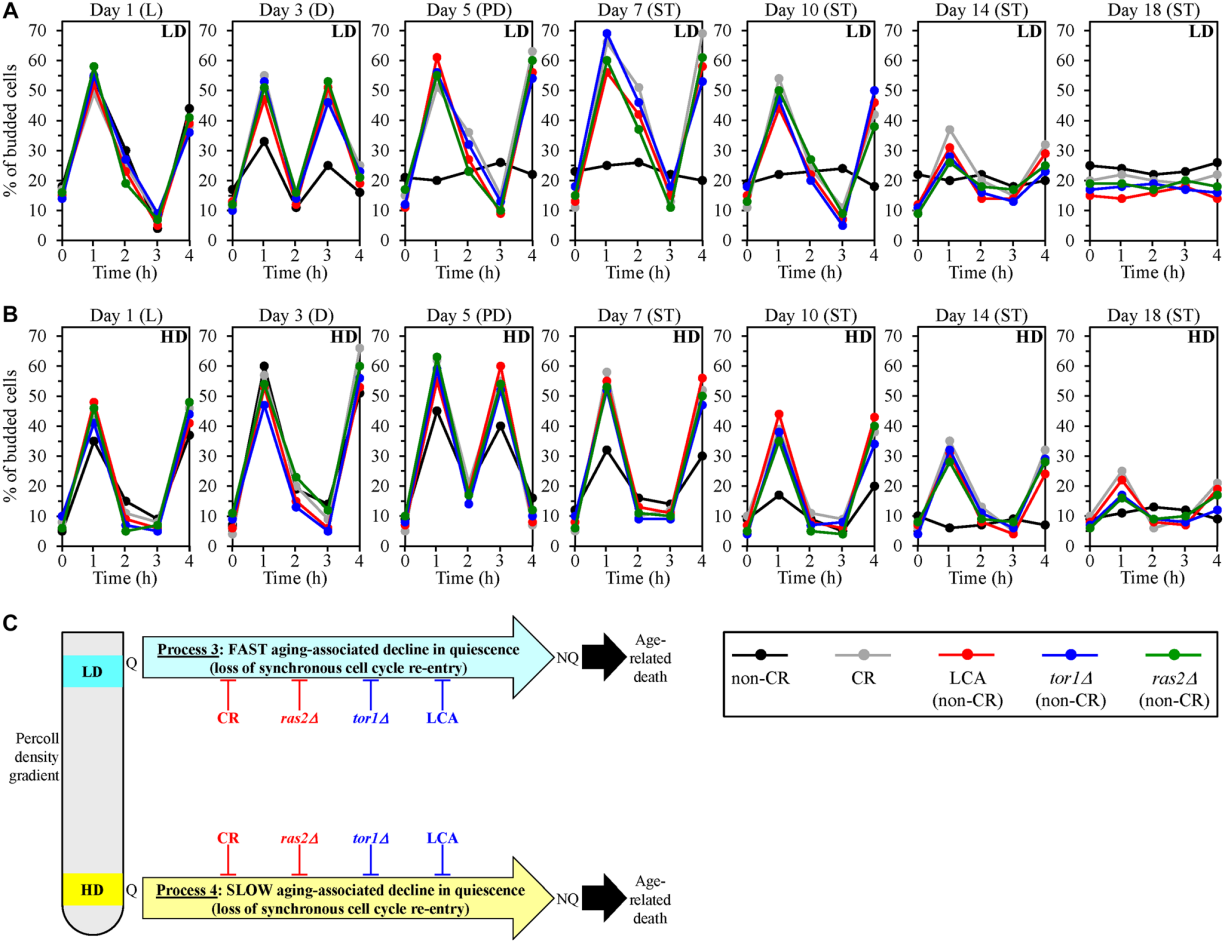
Caloric restriction (CR), lithocholic acid (LCA), the *tor1Δ* mutation and the *ras2Δ* mutation delay an aging-associated deterioration in the abilities of low-density quiescent (Q) cells and high-density Q cells to re-enter the mitotic cell cycle synchronously during processes 3 and 4, respectively. Wild-type (WT) yeast cells were cultured in the nutrient-rich YP medium initially containing 0.2% glucose (CR conditions) or 2% glucose (non-CR conditions) with or without LCA. The *tor1Δ* and *ras2Δ* mutant cells were cultured in the nutrient-rich YP medium initially containing 2% glucose (non-CR conditions) without LCA. Aliquots of differently aged cell populations were recovered from the logarithmic (L), diauxic (D), post-diauxic (PD) or stationary (ST) growth phase. High-density Q cells and low-density Q cells were purified from these cell populations with the help of the centrifugation in Percoll density gradient, as described in Materials and Methods. A detailed in Materials and Methods assay was used for assessing the abilities of low-density Q cells (**A**) and high-density Q cells (**B**) in differently aged WT or mutant cell populations to synchronously re-enter the mitotic cell cycle after cell transfer into fresh medium and incubation for 1 to 4 h. (**C**) A model for how CR, LCA (under non-CR conditions), *tor1Δ* (under non-CR conditions) and *ras2Δ* (under non-CR conditions) influence an aging-associated decline in the synchronous cell cycle re-entry for low-density Q cells and high-density Q cells during processes 3 and 4, respectively. Data in A and B are presented as means (*n* = 3). Other Abbreviations: HD: high-density cells; LD: low-density cells; NQ: non-quiescent cells.

We found that low-density Q cells in control non-CR cultures lose the ability to re-enter the mitotic cell cycle synchronously and become NQ cells since day 5 (the PD growth phase) of culturing (Figure 5A). Yet, the CR, LCA, *tor1Δ* and *ras2Δ* geroprotectors slowed down the fast aging-associated loss of such ability by low-density Q cells (Figure 5A).

We also found that high-density Q cells in control non-CR cultures cannot synchronously re-enter the mitotic cell cycle since day 14 (the ST growth phase) of culturing (Figure 5B). However, all four tested geroprotectors delayed the slow aging-associated loss of such ability by high-density Q cells (Figure 5B).

Thus, we concluded that CR, LCA, *tor1Δ* and *ras2Δ* slow down an aging-associated decline in the abilities of low-density Q cells and high-density Q cells to re-enter the mitotic cell cycle synchronously during processes 3 and 4, respectively (Figure 5C).

In sum, the findings presented in this section prove that the CR, LCA, *tor1Δ* and *ras2Δ* geroprotectors slow down the aging-associated process 3 of a fast deterioration in the quiescence of low-density Q cells (Figure 5C). Furthermore, each of these four geroprotectors also delays the aging-associated process 4 of a slow decline in the quiescence of high-density Q cells (Figure 5C).

### Two different ways of delaying yeast chronological aging by geroprotectors that differently affect the mechanism potentially linking cellular aging to cellular quiescence

**Figure 6:**
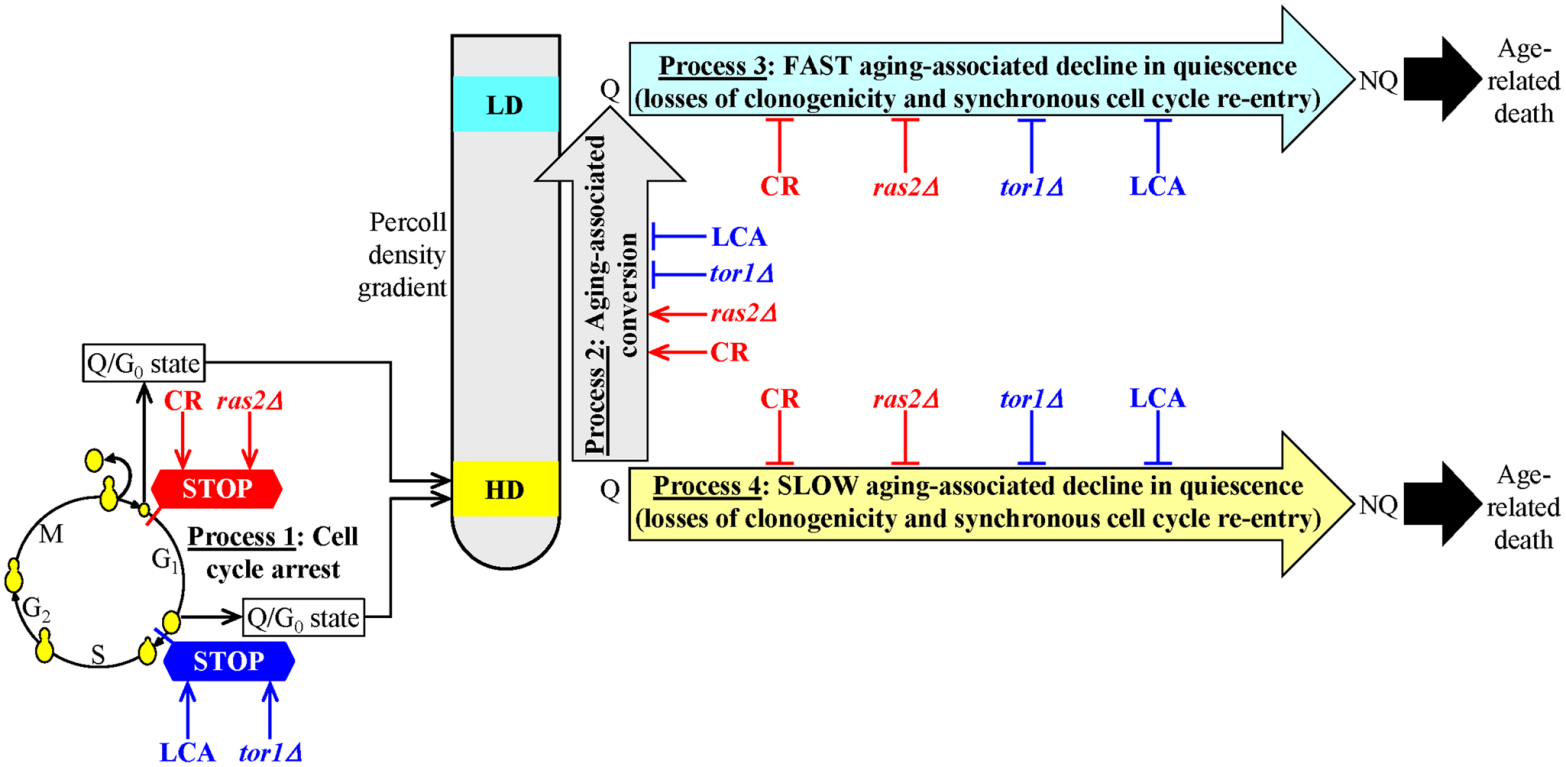
A model for the two different ways of delaying yeast chronological aging by geroprotectors that differently affect the mechanism potentially linking cellular aging to cellular quiescence. The first way is to initiate the formation of high-density quiescent (Q) cells (process 1) by arresting the cell cycle in early G_1_, speed up an age-related transition of high-density Q cells to low-density Q cells (process 2), slow down a fast aging-associated decline in the quiescence of low-density Q cells (process 3) and decelerate a slow aging-associated decline in the quiescence of high-density Q cells (process 4). Caloric restriction (CR) and the *ras2Δ* mutation (under non-CR conditions) postpone yeast chronological aging because they operate via the first way of targeting the mechanism that could link cellular aging to cellular quiescence. The second way is to promote the development of high-density Q cells (process 1) by arresting the cell cycle in late G_1_, postpone an age-related conversion of high-density Q cells into low-density Q cells (process 2), delay a fast aging-associated decline in the quiescence of low-density Q cells (process 3) and decelerate a slow aging-associated decline in the quiescence of high-density Q cells (process 4). Lithocholic acid (LCA) and the *tor1Δ* mutation (both under non-CR conditions) delay yeast chronological aging because they act via the second way of targeting the mechanism potentially linking cellular aging to cellular quiescence.

Our findings described in the previous three sections indicate that CR and the *ras2Δ* mutation (if present in yeast cultured under non-CR conditions) affect the mechanism that could link cellular aging to cellular quiescence in a different way than LCA and the *tor1Δ* mutation (if added to or present in yeast cultured under non-CR conditions, respectively). CR and the *ras2Δ* mutation initiate the formation of a stem cell niche of high-density Q cells (process 1) by arresting the cell cycle in early G_1_, whereas LCA and the *tor1Δ* mutation promote process 1 by arresting the cell cycle in late G_1_ (Figure 6). Furthermore, CR and the *ras2Δ* mutation speed up an age-related conversion of high-density Q cells into low-density Q cells (process 2), whereas LCA and the *tor1Δ* mutation slow down process 2 (Figure 6). Yet, each of the four tested geroprotective interventions similarly affects processes 3 and 4. Each of them decelerates a fast aging-associated decline in the quiescence of low-density Q cells (process 3) (Figure 6). They also postpone a slow aging-associated decline in the quiescence of high-density Q cells (process 4) (Figure 6).

Thus, there are the following two ways of slowing down yeast chronological aging by geroprotectors that differently affect processes 1, 2, 3 and 4.

The first way is to promote the formation of high-density Q cells (process 1) by arresting the cell cycle in early G_1_, accelerate an age-related conversion of high-density Q cells into low-density Q cells (process 2), slow down a fast aging-associated decline in the quiescence of low-density Q cells (process 3) and decelerate a slow aging-associated deterioration in the quiescence of high-density Q cells (process 4) (Figure 6). CR and the *ras2Δ* mutation postpone yeast chronological aging because they operate via the first way of targeting the mechanism potentially linking cellular aging to cellular quiescence (Figure 6).

The second way is to initiate the formation of high-density Q cells (process 1) by arresting the cell cycle in late G_1_, slow down an age-related conversion of high-density Q cells into low-density Q cells (process 2), postpone a fast aging-associated decline in the quiescence of low-density Q cells (process 3) and decelerate a slow aging-associated decline in the quiescence of high-density Q cells (process 4) (Figure 6). LCA and the *tor1Δ* mutation delay yeast chronological aging because they act via the second way of targeting the mechanism that could link cellular aging to cellular quiescence (Figure 6).

We selected CR and LCA to investigate the two ways different geroprotectors postpone yeast chronological aging by differently targeting the mechanism potentially linking cellular aging to cellular quiescence.

### A characteristic rise in glycogen within Q cells does not contribute either to aging delay by CR and LCA or to their effects on the mechanism potentially linking cellular aging to cellular quiescence

We hypothesized that certain metabolic traits of Q cells could play essential roles in the abilities of CR and LCA to postpone yeast chronological aging by differently affecting the mechanism that could link cellular aging to cellular quiescence. One such trait is increased glycogen concentration within Q cells under CR conditions of culturing (Figure 1A and 1B) [109]. Therefore, we first assessed how the single-gene deletion mutations that alter the intracellular glycogen concentration influence the extent of aging delay by CR and LCA. We also examined how these mutations affect the efficiencies of processes 1, 2, 3 and 4, all of which are integrated into the mechanism potentially linking cellular aging to cellular quiescence (Figure 1).

**Figure 7:**
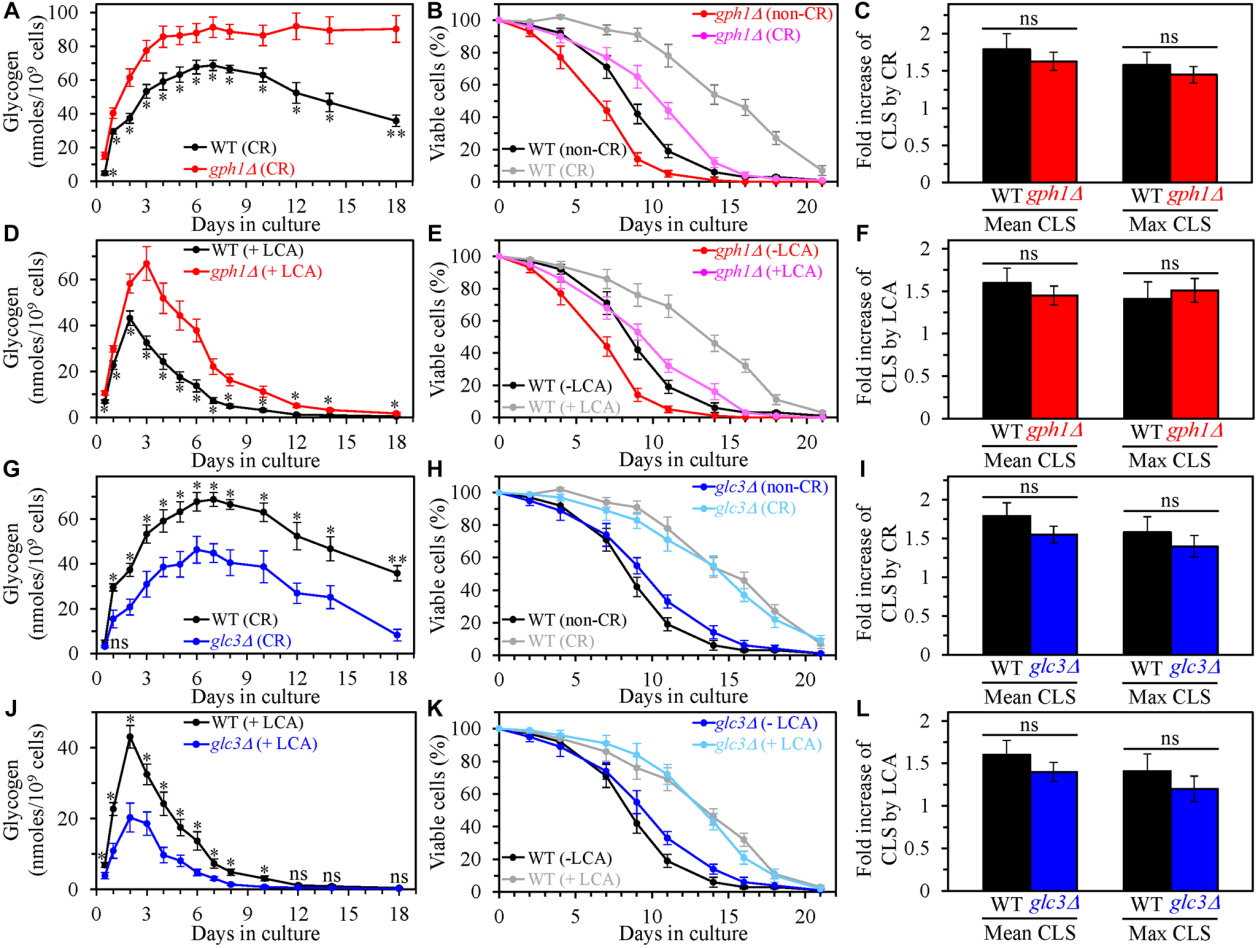
The single-gene deletion mutations that increase or decrease the intracellular glycogen concentration do not affect the efficiency of yeast chronological lifespan (CLS) extension by caloric restriction (CR) or lithocholic acid (LCA). Wild-type (WT), *gph1Δ* and *glc3Δ* cells were cultured in the nutrient-rich YP medium initially containing 0.2% glucose (CR conditions) or 2% glucose (non-CR conditions) with or without LCA. (**A**–**C**) The concentrations of glycogen in chronologically aging WT and *gph1Δ* cell cultures grown under CR conditions (A), survival curves of WT and *gph1Δ* cells cultured under CR or non-CR conditions (B), and the extent to which CR increases the mean and maximum CLS of WT and *gph1Δ* strains (C) are shown. (**D**–**F**) The concentrations of glycogen in chronologically aging WT and *gph1Δ* cell cultures grown under non-CR conditions with LCA (D), survival curves of WT and *gph1Δ* cells cultured under non-CR conditions with or without LCA (E), and the extent to which LCA under non-CR conditions increases the mean and maximum CLS of WT and *gph1Δ* strains (F) are shown. (**G**–**I**) The concentrations of glycogen in chronologically aging WT and *glc3Δ* cell cultures grown under CR conditions (G), survival curves of WT and *glc3Δ* cells cultured under CR or non-CR conditions (H), and the extent to which CR increases the mean and maximum CLS of WT and *glc3Δ* strains (I) are shown. (**J**–**L**) The concentrations of glycogen in chronologically aging WT and *glc3Δ* cell cultures grown under non-CR conditions with LCA (J), survival curves of WT and *glc3Δ* cells cultured under CR conditions without LCA or under non-CR conditions with LCA (K), and the extent to which LCA under non-CR conditions increases the mean and maximum CLS of WT and *glc3Δ* strains (L) are shown. Data are presented as means ± SEM (*n* = 3; ^*^ *p* < 0.05; ^**^ *p* < 0.01; ns, not significant; the *p* values for comparing the means of two in groups were calculated using an unpaired two-tailed *t* test as described in Materials and Methods).

The single-gene deletion mutation *gph1Δ* impairs glycogen debranching to glucose-1-phosphate because it prevents the removal of alpha-1,4-linked glucose units that precede an alpha-1,6-branch point [128–130]. However, it does not affect the Gdb1p-dependent debranching of this storage carbohydrate to glucose [130]. The *gph1Δ* mutation is known to increase the intracellular concentration of glycogen in yeast cultured under non-CR conditions [128]. We found that *gph1Δ* also elicits an age-related rise in glycogen concentration in yeast limited in calorie supply (Figure 7A) and yeast cultured under non-CR conditions in the presence of LCA (Figure 7D). It needs to be emphasized that, although the *gph1Δ* mutation shortened yeast CLS under CR conditions in the absence of LCA (Figure 7B) and non-CR conditions in the presence of LCA (Figure 7E), it did not alter the extent to which yeast CLS was extended by CR (Figure 7C) or by LCA (Figure 7F). Thus, the *gph1Δ*-dependent increase in glycogen concentration does not affect the efficiency of yeast CLS extension by CR or LCA.

Moreover, we found that the *gph1Δ* mutation has no significant effects on processes 1, 2, 3 and 4 (Figure 1) either in yeast cultured under CR conditions without LCA or in yeast cultured under non-CR conditions with LCA. This conclusion is based on the following observations.

**Figure 8:**
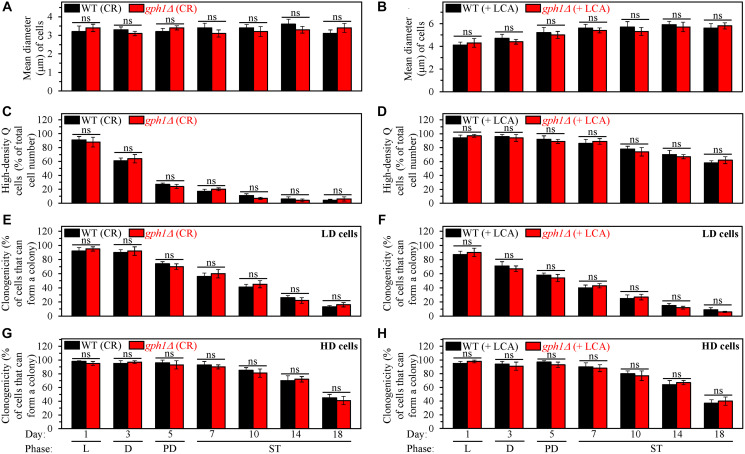
The *gph1Δ*-dependent increase in glycogen concentration does not affect processes 1, 2, 3 and 4 either in yeast cultured under caloric restriction (CR) conditions without LCA or in yeast cultured under non-CR conditions with lithocholic acid (LCA). Wild-type (WT) and *gph1Δ* cells were cultured in the nutrient-rich YP medium initially containing 0.2% glucose (CR conditions) or 2% glucose (non-CR conditions) with LCA. Aliquots of differently aged cell populations were recovered from the logarithmic (L), diauxic (D), post-diauxic (PD) or stationary (ST) growth phase. High-density quiescent (Q) cells and low-density Q cells were purified from these cell populations with the help of the centrifugation in Percoll density gradient, as described in Materials and Methods. (**A** and **B**) Mean diameters of high-density Q cells recovered from different growth phases are shown for yeast cultured under CR conditions without LCA (A) and yeast cultured under non-CR conditions with LCA (B). Mean diameters of high-density Q cells in A and B were measured using differential interference contrast microscopy and subsequent morphometric analysis, as described in Materials and Methods. (**C** and **D**) The values of the percentage of high-density Q cells in each of the differently aged cell populations are shown for yeast cultured under CR conditions without LCA (C) and yeast cultured under non-CR conditions with LCA (D). The percentage of high-density Q cells in C and D was measured with the help of a cell counter, as described in Materials and Methods. (**E**–**H**) The clonogenicities of low-density (E, F) and high-density (G, H) cells in each of the differently aged cell populations are shown for yeast cultured under CR conditions without LCA (E, G) and yeast cultured under non-CR conditions with LCA (F, H). The clonogenicities of low- and high-density cells were assessed using a plating assay for reproductive (colony forming) capability, as described in Materials and Methods. Other Abbreviations: HD: high-density cells; LD: low-density cells.

First, *gph1Δ* did not alter the size of high-density Q cells in yeast cultured under either of these two conditions. The high-density Q cells remained small in the *gph1Δ* strain under CR conditions without LCA (Figure 8A) and continued to be large in this mutant strain under non-CR conditions with LCA (Figure 8B). Based on the observed sizes of high-density Q cells, we concluded that the *gph1Δ*-dependent increase in glycogen concentration does not affect the ability of CR to initiate the formation of small high-density Q cells (process 1) by arresting the cell cycle and causing entry into quiescence at a checkpoint in early G_1_. We also concluded that the *gph1Δ*-driven rise in glycogen concentration does not influence the ability of LCA to promote the formation of large high-density Q cells under non-CR conditions (process 1) by causing the cell cycle arrest and quiescence entry at a checkpoint in late G_1_.

Second, *gph1Δ* had no significant effect on the fast, age-related conversion of high-density Q cells into low-density Q cells (process 2) under CR conditions without LCA (Figure 8C). This mutation also did not influence the slow, age-related transition from high-density Q cells to low-density Q cells (process 2) under non-CR conditions with LCA (Figure 8D). Thus, the *gph1Δ*-dependent increase in glycogen concentration does not influence either the ability of CR to speed up process 2 or the ability of LCA to slow down this process under non-CR conditions.

Third, *gph1Δ* did not influence the extent of the fast, aging-associated decline in the clonogenicity of low-density Q cells (process 3) either under CR conditions in the absence of LCA (Figure 8E) or under non-CR conditions in the presence of LCA (Figure 8F). Also, *gph1Δ* did not affect the efficiency of the slow, aging-associated deterioration in the clonogenicity of high-density Q cells (process 4) under any of these two conditions (Figure 8G and 8H). Therefore, we concluded that the *gph1Δ*-dependent increase in glycogen concentration has no significant effect on the abilities of CR and LCA to delay a decline in cellular quiescence during processes 3 and 4.

We then assessed the effects of the single-gene deletion mutation *glc3Δ* on the extent of aging delay by CR and LCA and processes 1, 2, 3 and 4. This mutation impairs a branching step of glycogen biosynthesis and decreases glycogen concentration in *S. cerevisiae* cells cultured under non-CR conditions [131, 132]. We found that *glc3Δ* also causes an age-related decline in glycogen concentration in yeast cultured under CR conditions without LCA (Figure 7G) or under non-CR conditions with LCA (Figure 7J). Despite a minor rise in yeast CLS elicited by *glc3Δ* under CR conditions without LCA (Figure 7H) and non-CR conditions with LCA (Figure 7K), this mutation did not affect the efficiency of the CLS extension under either of these two conditions of cell culturing (Figure 7I and 7L, respectively). Therefore, we concluded that the *glc3Δ*-dependent decline in glycogen concentration does not influence the CR- or LCA-driven longevity extension of chronologically aging yeast.

Our findings also indicate that the *glc3Δ* mutation does not affect processes 1, 2, 3 and 4 (Figure 1) either in yeast cultured under CR conditions in the absence of LCA or yeast cultured under non-CR conditions in the presence of LCA. These findings are described below.

**Figure 9:**
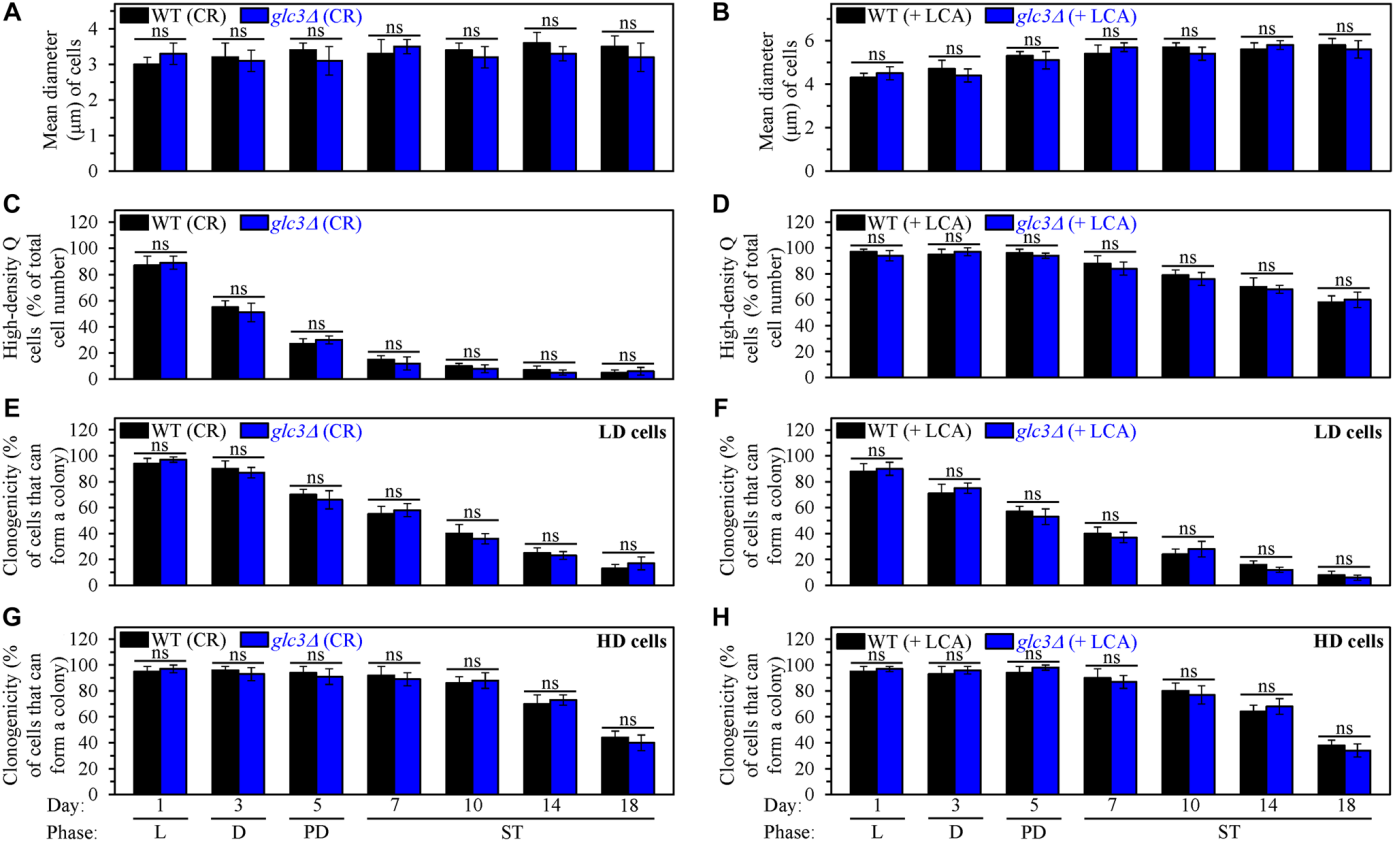
The *glc3Δ*-dependent decline in glycogen concentration does not affect processes 1, 2, 3 and 4 either in yeast cultured under caloric restriction (CR) conditions without LCA or in yeast cultured under non-CR conditions with lithocholic acid (LCA). Wild-type (WT) and *glc3Δ* cells were cultured in the nutrient-rich YP medium initially containing 0.2% glucose (CR conditions) or 2% glucose (non-CR conditions) with LCA. Aliquots of differently aged cell populations were recovered from the logarithmic (L), diauxic (D), post-diauxic (PD) or stationary (ST) growth phase. High-density quiescent (Q) cells and low-density Q cells were purified from these cell populations with the help of the centrifugation in Percoll density gradient, as described in Materials and Methods. (**A** and **B**) Mean diameters of high-density Q cells recovered from different growth phases are shown for yeast cultured under CR conditions without LCA (A) and yeast cultured under non-CR conditions with LCA (B). Mean diameters of high-density Q cells in A and B were measured using differential interference contrast microscopy and subsequent morphometric analysis, as described in Materials and Methods. (**C** and **D**) The values of the percentage of high-density Q cells in each of the differently aged cell populations are shown for yeast cultured under CR conditions without LCA (C) and yeast cultured under non-CR conditions with LCA (D). The percentage of high-density Q cells in C and D was measured with the help of a cell counter, as described in Materials and Methods. (**E**–**H**) The clonogenicities of low-density (E, F) and high-density (G, H) cells in each of the differently aged cell populations are shown for yeast cultured under CR conditions without LCA (E, G) and yeast cultured under non-CR conditions with LCA (F, H). The clonogenicities of low- and high-density cells were assessed using a plating assay for reproductive (colony forming) capability, as described in Materials and Methods. Other Abbreviations: HD: high-density cells; LD: low-density cells.

First, a comparison of sizes of high-density Q cells indicates that the *glc3Δ*-dependent decline in glycogen concentration does not influence the ability of CR to arrest the cell cycle at a checkpoint in early G_1_, thereby promoting quiescence entry and eliciting the development of small high-density Q cells (process 1) (Figure 9A). The comparison of sizes of high-density Q cells also shows that the *glc3Δ*-driven decrease in glycogen concentration does not affect the ability of LCA to arrest the cell cycle at a checkpoint in late G_1_, thus initiating quiescence entry and causing the formation of large high-density Q cells under non-CR conditions (process 1) (Figure 9B).

Second, a comparison of the efficiencies with which high-density Q cells are converted into low-density Q cells (process 2) in an age-related manner shows that the *glc3Δ* mutation does not affect this process either under CR conditions without LCA (Figure 9C) or under non-CR conditions with LCA (Figure 9D). Hence, the *glc3Δ*-dependent decline in glycogen concentration has no effect either on the acceleration of process 2 by CR or on the deceleration of this process by LCA under non-CR conditions.

Third, we also investigated the effects of the *glc3Δ* mutation on the fast, aging-associated deterioration in the clonogenicity of low-density Q cells (process 3) and slow, aging-associated decline in the clonogenicity of high-density Q cells (process 4). We found that *glc3Δ* does not affect processes 3 and 4 either under CR conditions without LCA (Figure 9E and 9G, respectively) or under non-CR conditions with LCA (Figure 9F and 9H, respectively). Based on these findings, we concluded that the *glc3Δ*-driven reduction in glycogen concentration has no effect on the slowing down of these processes either by CR or by LCA under non-CR conditions.

Collectively, the findings described in this section provide evidence that an increase in the intracellular concentration of glycogen, which is a specific metabolic trait of Q cells, is not essential for the extension of yeast CLS by CR and LCA. These findings also show that a characteristic rise in glycogen within Q cells is not an essential contributor to the effects of CR and LCA on the mechanism potentially linking cellular aging to cellular quiescence.

### An increase in trehalose concentration, a metabolic hallmark of Q cells, is an essential contributor to aging delay by CR and LCA and affects the mechanism that could link cellular aging to cellular quiescence

A CR-dependent rise in trehalose concentration is a distinct trait of Q cells (Figure 1A and 1B) [109]. We sought to investigate the role of this Q cell-specific metabolic trait in the delay of yeast chronological aging by CR and LCA. We also wondered if the CR-driven trehalose rise within Q cells may contribute to the specific process (or processes) integrated into the mechanism linking cellular aging to cellular quiescence. To address these challenges, we examined the effects of the single-gene deletion mutations that differently influence the intracellular concentration of trehalose on the efficiencies with which CR and LCA postpone chronological aging in *S. cerevisiae*. We also assessed how these mutations influence processes 1, 2, 3 and 4, all of which are involved in the mechanism that could link cellular aging to cellular quiescence (Figure 1).

**Figure 10:**
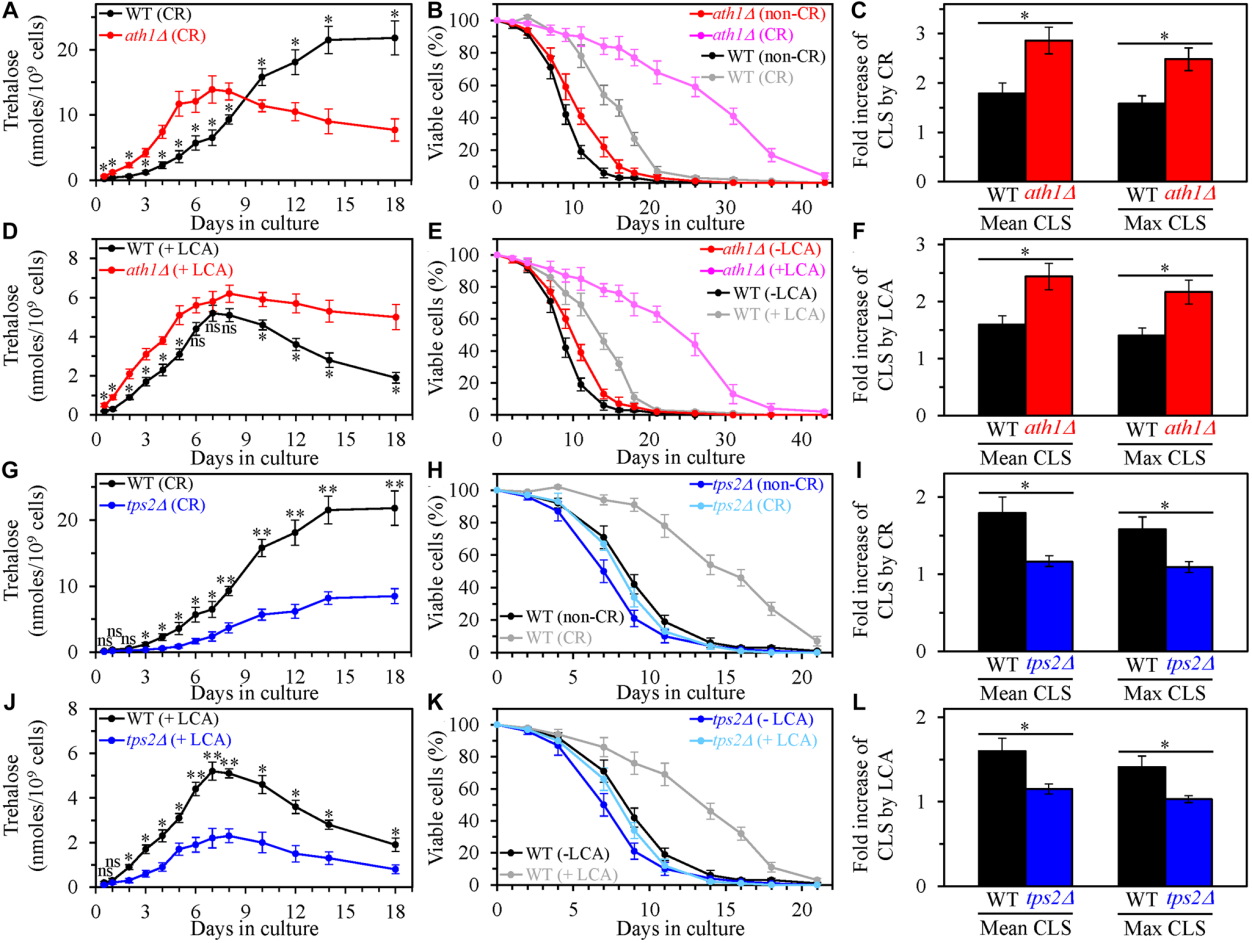
The single-gene deletion mutations that increase or decrease the intracellular concentration of trehalose alter the extent to which caloric restriction (CR) and lithocholic acid (LCA) prolong yeast chronological lifespan (CLS). Wild-type (WT), ath1*Δ* and tps2*Δ* cells were cultured in the nutrient-rich YP medium initially containing 0.2% glucose (CR conditions) or 2% glucose (non-CR conditions) with or without LCA. (**A**–**C**) The concentrations of trehalose in chronologically aging WT and *ath1Δ* cell cultures grown under CR conditions (A), survival curves of WT and *ath1Δ* cells cultured under CR or non-CR conditions (B), and the extent to which CR increases the mean and maximum CLS of WT and *ath1Δ* strains (C) are shown. (**D**–**F**) The concentrations of trehalose in chronologically aging WT and *ath1Δ* cell cultures grown under non-CR conditions with LCA (D), survival curves of WT and *ath1Δ* cells cultured under non-CR conditions with or without LCA (E), and the extent to which LCA under non-CR conditions increases the mean and maximum CLS of WT and *ath1Δ* strains (F) are shown. (**G**–**I**) The concentrations of trehalose in chronologically aging WT and *tps2Δ* cell cultures grown under CR conditions (G), survival curves of WT and *tps2Δ* cells cultured under CR or non-CR conditions (H), and the extent to which CR increases the mean and maximum CLS of WT and *tps2Δ* strains (I) are shown. (**J**–**L**) The concentrations of trehalose in chronologically aging WT and *tps2Δ* cell cultures grown under non-CR conditions with LCA (J), survival curves of WT and *tps2Δ* cells cultured under CR conditions without LCA or under non-CR conditions with LCA (K), and the extent to which LCA under non-CR conditions increases the mean and maximum CLS of WT and *glc3Δ* strains (L) are shown. Data are presented as means ± SEM (*n* = 3; ^*^ *p* < 0.05; ^**^ *p* < 0.01; ns, not significant; the *p* values for comparing the means of two in groups were calculated using an unpaired two-tailed *t* test as described in Materials and Methods).

The single-gene deletion mutation *ath1Δ* impairs intracellular and extracellular trehalose degradation into two glucose molecules by the acid trehalase enzyme Ath1 [133–135]. This mutation has been shown to raise the intracellular concentration of trehalose in yeast cultured under non-CR conditions [135]. Our assessment of intracellular trehalose in yeast cultured under CR conditions revealed that *ath1Δ* increases trehalose concentration during the L, D and PD growth phases but has an opposite effect on intracellular trehalose during the ST growth phase (Figure 10A) [136]. We also found that the *ath1Δ* mutation causes a rise in trehalose concentration during all these growth phases (*i.e.*, through the entire CLS) in yeast cultured under non-CR conditions in the presence of LCA (Figure 10D). Noteworthy, *ath1Δ* extended yeast CLS under CR conditions in the absence of LCA (Figure 10B) and increased the longevity-extending efficiency of CR in chronologically aging yeast (Figure 10B and 10C). Besides, *ath1Δ* prolonged yeast CLS under non-CR conditions in the presence of LCA (Figure 10E) and amplified the efficiency of yeast CLS extension by LCA under these conditions (Figure 10E and 10F). Based on these findings, we concluded that the *ath1Δ*-dependent changes in trehalose concentration through the entire CLS define the efficiency of yeast CLS extension by CR. We also deduced that the *ath1Δ*-driven rise in trehalose concentration through the entire CLS enhances the ability of LCA to prolong yeast CLS under non-CR conditions.

We then examined the effects of the *ath1Δ* mutation on the efficiencies of processes 1, 2, 3 and 4, all of which converge into the mechanism potentially linking cellular aging to cellular quiescence (Figure 1). We found that *ath1Δ* does not affect process 1 but significantly affects three other processes. Findings that support our conclusion are described below.

**Figure 11:**
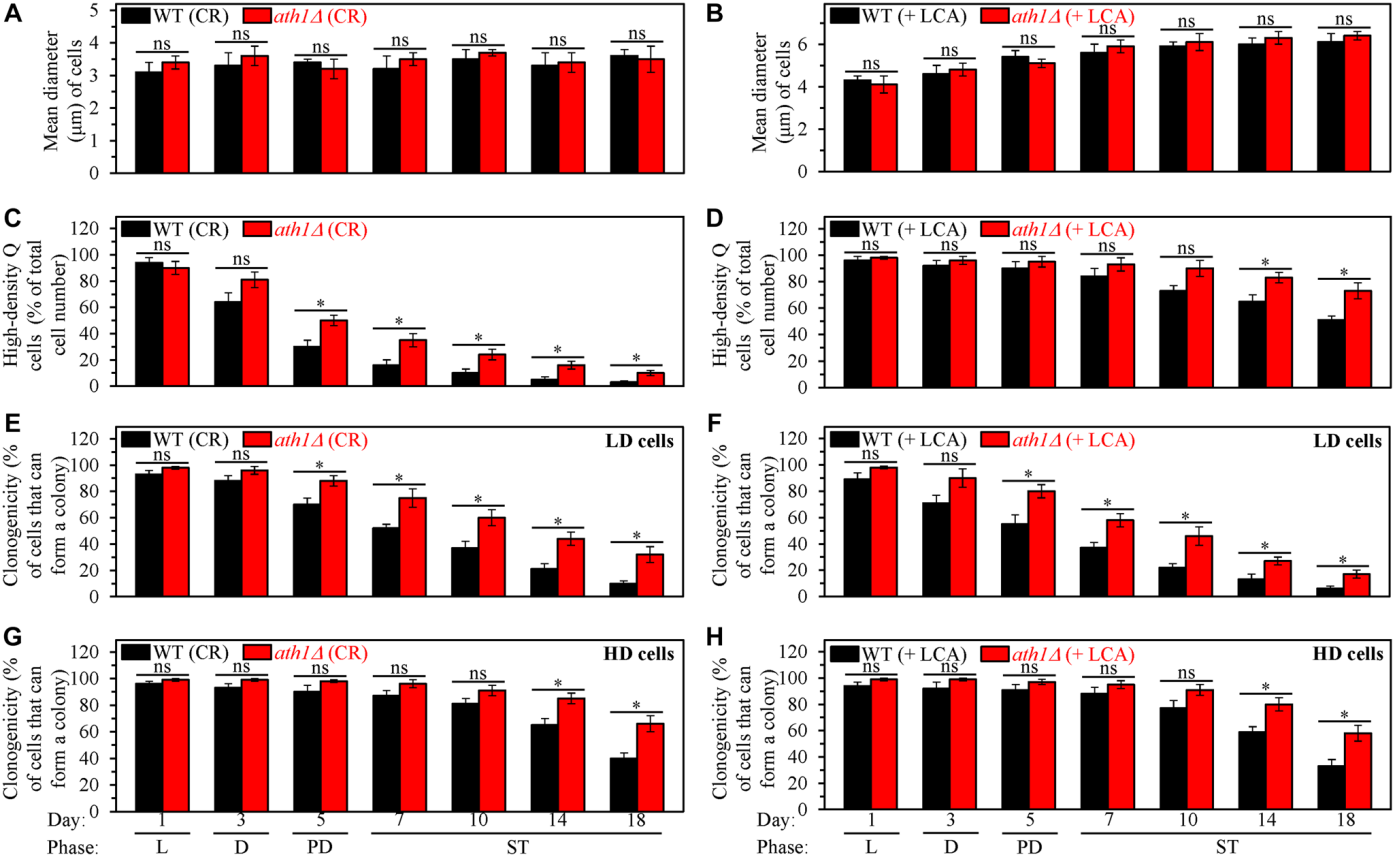
The *ath1Δ*-dependent changes in trehalose concentration do not affect process 1 but exhibit significant effects on processes 2, 3 and 4 both in yeast cultured under caloric restriction (CR) conditions without LCA and in yeast cultured under non-CR conditions with lithocholic acid (LCA). Processes 1, 2, 3 and 4 converge into the mechanism that could link cellular aging to cellular quiescence. Wild-type (WT) and *ath1Δ* cells were cultured in the nutrient-rich YP medium initially containing 0.2% glucose (CR conditions) or 2% glucose (non-CR conditions) with LCA. Aliquots of differently aged cell populations were recovered from the logarithmic (L), diauxic (D), post-diauxic (PD) or stationary (ST) growth phase. High-density quiescent (Q) cells and low-density Q cells were purified from these cell populations with the help of the centrifugation in Percoll density gradient, as described in Materials and Methods. (**A** and **B**) Mean diameters of high-density Q cells recovered from different growth phases are shown for yeast cultured under CR conditions without LCA (A) and yeast cultured under non-CR conditions with LCA (B). Mean diameters of high-density Q cells in A and B were measured using differential interference contrast microscopy and subsequent morphometric analysis, as described in Materials and Methods. (**C** and **D**) The values of the percentage of high-density Q cells in each of the differently aged cell populations are shown for yeast cultured under CR conditions without LCA (C) and yeast cultured under non-CR conditions with LCA (D). The percentage of high-density Q cells in C and D was measured with the help of a cell counter, as described in Materials and Methods. (**E**–**H**) The clonogenicities of low-density (E, F) and high-density (G, H) cells in each of the differently aged cell populations are shown for yeast cultured under CR conditions without LCA (E, G) and yeast cultured under non-CR conditions with LCA (F, H). The clonogenicities of low- and high-density cells were assessed using a plating assay for reproductive (colony forming) capability, as described in Materials and Methods. Other Abbreviations: HD: high-density cells; LD: low-density cells.

First, the high-density Q cells limited in calorie supply and cultured in the absence of LCA were as small in the *ath1Δ* mutant strain as they were in the WT strain cultured under the same conditions (Figure 11A). Thus, the *ath1Δ*-dependent changes in trehalose concentrations do not affect the ability of CR to initiate the formation of small high-density Q cells (process 1) by arresting the cell cycle and eliciting entry into quiescence in early G_1_. The *ath1Δ* mutation also did not alter the size of the high-density Q cells cultured under non-CR conditions with LCA; these cells in the mutant strain remained as large as they were in the WT strain under the same culturing conditions (Figure 11B). Hence, the *ath1Δ*-driven rise in trehalose concentration does not influence the ability of LCA to arrest the cell cycle in late G_1_, instigate quiescence entry and cause the formation of large high-density Q cells under non-CR conditions (process 1).

Second, *ath1Δ* slowed down the fast, age-related transition from high-density Q cells to low-density Q cells (process 2) under CR conditions without LCA (Figure 11C). This mutation also decelerated the slow, age-related conversion of high-density Q cells into low-density Q cells (process 2) under non-CR conditions with LCA (Figure 11D). Based on these findings, we concluded that the *ath1Δ*-dependent changes in trehalose concentration contribute to both the acceleration of process 2 by CR and the deceleration of this process by LCA under non-CR conditions.

Third, *ath1Δ* decelerated the fast, aging-associated decline in the clonogenicity of low-density Q cells (process 3) both under CR conditions in the absence of LCA (Figure 11E) and under non-CR conditions in the presence of LCA (Figure 11F). Moreover, *ath1Δ* delayed the slow, aging-associated deterioration in the clonogenicity of high-density Q cells (process 4) under both these conditions of cell culturing (Figure 11G and 11H, respectively). Therefore, we concluded that the *ath1Δ*-driven changes in trehalose concentration play essential roles in the abilities of CR and LCA to slow down an age-related decline in cellular quiescence during processes 3 and 4.

We then assessed the effects of the single-gene deletion mutation *tps2Δ* on the extent of aging delay by CR and LCA and processes 1, 2, 3 and 4. This mutation impairs intracellular trehalose synthesis by eliminating a phosphatase subunit of the trehalose-6-phosphate synthase/phosphatase complex [130, 137]. *tps2Δ* is known to decrease trehalose concentration in *S. cerevisiae* cells cultured under non-CR conditions [137]. We found that *tps2Δ* also elicits a decline in trehalose concentration throughout the entire CLS in yeast cultured under CR conditions without LCA (Figure 10G) or under non-CR conditions with LCA (Figure 10J). Our investigation of the effects of the *tps2Δ* mutation on longevity revealed that it shortens yeast CLS under CR conditions without LCA (Figure 10H) and significantly lowers the efficiency of CLS extension by CR (Figure 10H and 10I). We also found that *tps2Δ* reduces yeast CLS under non-CR conditions with LCA (Figure 10K) and substantially decreases the extent to which LCA postpones yeast CLS under these conditions (Figure 10K and 10L). Hence, the *tps2Δ*-dependent decline in trehalose concentration through the entire CLS lowers the efficiencies of yeast CLS extension both by CR and LCA (under non-CR conditions).

Our findings also show that the *tps2Δ* mutation does not influence process 1. However, it affects processes 2, 3 and 4, all of which are integrated into the mechanism linking cellular aging to cellular quiescence (Figure 1). These conclusions are based on the observations described below.

**Figure 12:**
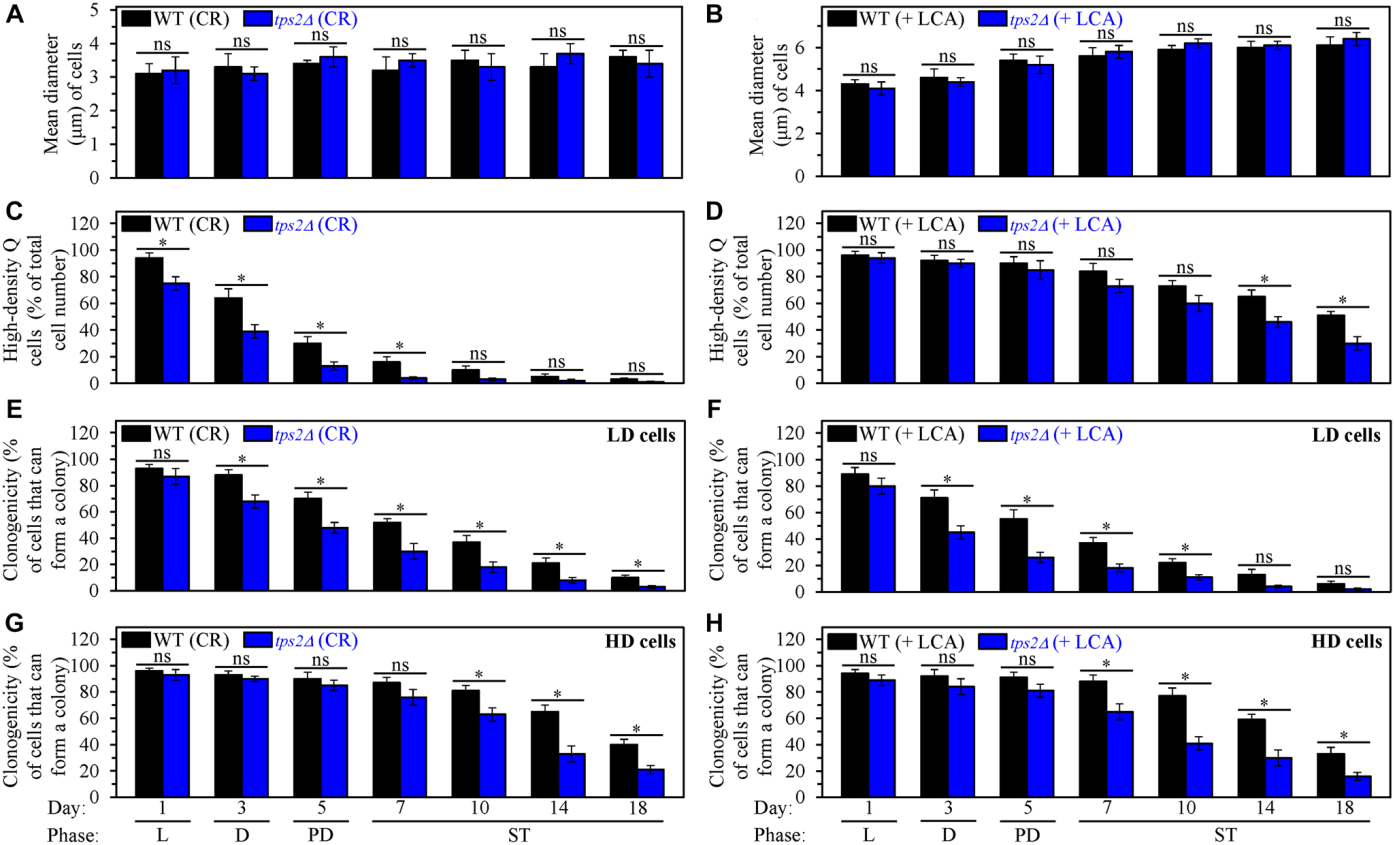
The *tps2Δ*-driven decline in trehalose concentration does not affect process 1 but exhibits significant effects on processes 2, 3 and 4 both in yeast cultured under caloric restriction (CR) conditions without LCA and in yeast cultured under non-CR conditions with lithocholic acid (LCA). Processes 1, 2, 3 and 4 converge into the mechanism that could link cellular aging to cellular quiescence. Wild-type (WT) and *tps2Δ* cells were cultured in the nutrient-rich YP medium initially containing 0.2% glucose (CR conditions) or 2% glucose (non-CR conditions) with LCA. Aliquots of differently aged cell populations were recovered from the logarithmic (L), diauxic (D), post-diauxic (PD) or stationary (ST) growth phase. High-density quiescent (Q) cells and low-density Q cells were purified from these cell populations with the help of the centrifugation in Percoll density gradient, as described in Materials and Methods. (**A** and **B**) Mean diameters of high-density Q cells recovered from different growth phases are shown for yeast cultured under CR conditions without LCA (A) and yeast cultured under non-CR conditions with LCA (B). Mean diameters of high-density Q cells in A and B were measured using differential interference contrast microscopy and subsequent morphometric analysis, as described in Materials and Methods. (**C** and **D**) The values of the percentage of high-density Q cells in each of the differently aged cell populations are shown for yeast cultured under CR conditions without LCA (C) and yeast cultured under non-CR conditions with LCA (D). The percentage of high-density Q cells in C and D was measured with the help of a cell counter, as described in Materials and Methods. (**E**–**H**) The clonogenicities of low-density (E, F) and high-density (G, H) cells in each of the differently aged cell populations are shown for yeast cultured under CR conditions without LCA (E, G) and yeast cultured under non-CR conditions with LCA (F, H). The clonogenicities of low- and high-density cells were assessed using a plating assay for reproductive (colony forming) capability, as described in Materials and Methods. Other Abbreviations: HD: high-density cells; LD: low-density cells.

First, we found that the *tps2Δ*-dependent decline in intracellular trehalose does not affect the abilities of CR and LCA (under non-CR conditions) to regulate process 1. This process leads to cell-cycle arrest, thus initiating quiescence entry and eliciting the formation of high-density Q cells (Figure 1). Indeed, we found that *tps2Δ* does not influence the ability of CR to arrest the cell cycle in early G_1_, which is known to promote the development of small high-density Q cells (Figure 12A). Moreover, we also revealed that *tps2Δ* does not affect the ability of LCA to arrest the cell cycle in late G_1_, which is known to cause the formation of large high-density Q cells under non-CR conditions (Figure 12B).

Second, we found that *tps2Δ* accelerates both the fast, age-related conversion of high-density Q cells into low-density Q cells (process 2) under CR conditions without LCA (Figure 12C) and the slow, age-related transition from high-density Q cells to low-density Q cells (process 2) under non-CR conditions with LCA (Figure 12D). Hence, the *tps2Δ*-dependent decline in trehalose concentration affects both the acceleration of process 2 by CR and the deceleration of this process by LCA under non-CR conditions.

Third, we found that *tps2Δ* speeds up both the fast, aging-associated deterioration in the clonogenicity of low-density Q cells (process 3) and the slow, aging-associated decline in the clonogenicity of high-density Q cells (process 4). These effects of the *tps2Δ* mutation on processes 3 and 4 were observed both under CR conditions in the absence of LCA (Figure 12E and 12G, respectively) and under non-CR conditions in the presence of LCA (Figure 12F and 12H, respectively). Thus, the *tps2Δ*-driven deterioration in trehalose concentration impairs the abilities of CR and LCA to decelerate an age-related decline in cellular quiescence during processes 3 and 4.

In sum, the data presented in this section show that a rise in intracellular trehalose, which is a metabolic hallmark of Q cells, contributes to the extension of yeast CLS by CR and LCA. Besides, these data indicate that such a rise in trehalose within Q cells plays essential roles in processes 2, 3 and 4, all of which converge into the mechanism potentially linking cellular aging to cellular quiescence.

## DISCUSSION

This study and our previously published data [109] provide conclusive evidence for the existence of a mechanism that links cellular aging to cellular quiescence in chronologically aging *S. cerevisiae*. The mechanism integrates processes 1, 2, 3 and 4 discussed above in the text and schematically depicted in Figures 1 and 6. Two lines of evidence that support our conclusion about the existence of this mechanism are discussed below.

The first line of evidence comes from our observation that all four aging-delaying interventions we tested affect the four processes converged into the mechanism linking cellular aging to cellular quiescence. These geroprotective interventions include CR, LCA and the *tor1Δ* and *ras2Δ* mutations. As discussed above in the text, there are two different ways of delaying yeast chronological aging by geroprotectors affecting processes 1, 2, 3 and 4. CR and the *ras2Δ* mutation decelerate yeast chronological aging because they both stimulate the development of high-density Q cells (process 1) by arresting the cell cycle in early G_1_, promote an age-related conversion of high-density Q cells into low-density Q cells (process 2), delay a fast aging-associated deterioration in the quiescence of low-density Q cells (process 3) and postpone a slow aging-associated decline in the quiescence of high-density Q cells (process 4) (Figure 6). Yet, LCA and the *tor1Δ* mutation delay yeast chronological aging because they both promote the development of high-density Q cells (process 1) by arresting the cell cycle in late G_1_, postpone an age-related conversion of high-density Q cells into low-density Q cells (process 2), slow down a fast aging-associated deterioration in the quiescence of low-density Q cells (process 3) and delay a slow aging-associated decline in the quiescence of high-density Q cells (process 4) (Figure 6).

Therefore, all four geroprotectors tested in this study exhibit a common ability to postpone processes 3 and 4 of the fast and slow quiescence deterioration for low- and high-density Q cells, respectively (Figure 6). Such a common ability of diverse geroprotectors provides strong support for the existence of a mechanism that links cellular aging to cellular quiescence in chronologically aging budding yeast. Indeed, this ability might contribute to the geroprotector-driven delay of yeast chronological aging because it allows Q cells to maintain a pro-longevity cellular pattern longer than Q cells in untreated yeast cultures. This pro-longevity cellular pattern of Q cells includes an enhanced reproductive competence, an increase in glycogen and trehalose concentrations, a decrease in the concentrations of TAG, a rise in CL concentrations, a decline in the concentrations of ROS, an improvement of mitochondrial functionality, lowered oxidative damage to macromolecules, a rise in cell resistance to long-term thermal and oxidative stresses, and a deterioration in cell susceptibility to apoptotic and liponecrotic forms of regulated cell death (Figure 1A and 1B) [109].

A challenge for the future is to investigate and understand how important the abilities of diverse geroprotectors affect processes 1 and 2 for their ability to postpone yeast chronological aging. One possibility is that the different ways through which diverse geroprotectors regulate processes 1 and 2 do not contribute to the aging-delaying capabilities of these geroprotectors. An alternative possibility is that there are two different ways of employing geroprotector-dependent changes in processes 1 and 2 to decelerate yeast chronological aging.

The second line of evidence for the existence of a mechanism linking cellular aging to cellular quiescence comes from our observation that an increase in intracellular trehalose within Q cells is an essential contributor to both chronological aging and quiescence maintenance in *S. cerevisiae*. We have previously demonstrated that such an increase in trehalose is a metabolic trait characteristic of Q cells in budding yeast [109]. In this study, we found that mutations altering trehalose concentration affect the extent of yeast CLS extension by CR or LCA and the efficiencies of processes 2, 3 and 4 in yeast exposed to any of these two geroprotectors. Future studies will need to examine what other hallmarks of Q cells (Figure 1A and 1B) play essential roles in yeast chronological aging and yeast quiescence maintenance.

In conclusion, because the mechanisms of cellular aging and cellular quiescence are evolutionarily conserved [1, 3, 62, 66], this study makes an important next step toward the understanding of how the knowledge-based targeting of cellular quiescence can be used for slowing down cellular and organismal aging and for delaying the onset of aging-associated diseases.

## MATERIALS AND METHODS

### Yeast strains, media and growth conditions

The wild-type strain *Saccharomyces cerevisiae* BY4742 (*MATα his3Δ1 leu2Δ0 lys2Δ0 ura3Δ0*) and single-gene-deletion mutant strains in the BY4742 genetic background (all from Thermo Scientific/Open Biosystems) were grown in YP medium (1% yeast extract, 2% peptone, both from Fisher Scientific; #BP1422-2 and #BP1420-2, respectively) initially containing 0.2% or 2% glucose (#D16-10; Fisher Scientific) as carbon source, with or without 50 μM lithocholic acid. Cells were cultured at 30°C with rotational shaking at 200 rpm in Erlenmeyer flasks at a “flask volume/medium volume” ratio of 5:1.

### Separation of quiescent and non-quiescent cells by centrifugation in Percoll density gradient

1 ml of 1.5 M NaCl (#S7653; Sigma-Aldrich) was placed into a 50-ml conical polypropylene centrifuge tube (#055398; Fisher Scientific), and 8 ml of the Percoll solution (#P1644; Sigma-Aldrich) was added to this tube. The NaCl and Percoll solutions were then mixed by pipetting. 4 ml of the NaCl/Percoll mixture was placed into each of the two polyallomer tubes for an MLS-50 rotor for an Optima MAX ultracentrifuge (all from Beckman Coulter, Inc.). The tubes were centrifuged at 25,000 × g (16,000 rpm) for 15 min at 4°C in an Optima MAX ultracentrifuge. A sample of yeast cells was taken from a culture at a certain time point. A sample fraction was diluted to determine the total number of cells per ml of culture using a hemacytometer (#0267110; Fisher Scientific). For each Percoll density gradient, 1 × 10^9^ yeast cells were placed into a 15-ml conical polypropylene centrifuge tube (#0553912; Fisher Scientific) and then pelleted by centrifugation at 5,000 rpm for 7 min at room temperature in an IEC Centra CL2 clinical centrifuge (Thermo Electron Corporation). Pelleted cells were resuspended in 500 μl of 50 mM Tris/HCl buffer (pH 7.5), overlaid onto the preformed Percoll gradient and centrifuged at 2,300 × g (5,000 rpm) for 30 min at 25°C in an Optima MAX ultracentrifuge. The upper and lower fractions of cells were collected with a pipette, Percoll was removed by washing cells twice with 50 mM Tris/HCl buffer (pH 7.5) and cells were resuspended in 50 mM Tris/HCl buffer (pH 7.5) for subsequent assays.

### Reproductive (colony-forming) capability assay for quiescent and non-quiescent cells separated by centrifugation in Percoll density gradient

An aliquot of the upper or lower fraction of cells recovered from the Percoll gradient and washed twice with 50 mM Tris/HCl buffer (pH 7.5) was diluted to determine the total number of cells per fraction using a hemacytometer (#0267110; Fisher Scientific). Serial dilutions (1:10^2^ to 1:10^5^) of cells were also plated onto YEPD (1% yeast extract, 2% peptone, 2% glucose) plates in duplicate to count the number of viable cells per ml of each cell fraction. 100 μl of diluted culture was plated onto each plate. After 48-h incubation at 30°C, the number of colonies per plate was counted. The number of colony-forming units (CFU) equals the number of reproductively capable cells in a sample. Therefore, the number of reproductively capable cells was calculated as follows: the number of CFU × dilution factor × 10 = the number of reproductively capable cells per ml. For each cell fraction assayed, the % reproductive capability of the cells was calculated as follows: number of CFU per ml/total number of cells per ml × 100%.

### Synchronous reentry into mitosis assay for quiescent and non-quiescent cells separated by centrifugation in Percoll density gradient

5 × 10^6^ cells recovered in the upper or lower fraction of the Percoll gradient and washed twice with 50 mM Tris/ HCl buffer (pH 7.5) were harvested by centrifugation for 1 min at 21,000 × g at room temperature. Pelleted cells were washed twice with water and then inoculated into 50 ml of YP medium (1% yeast extract, 2% peptone; both from Fisher Scientific; #BP1422-2 and #BP1420- 2, respectively) initially containing 0.2% or 2% glucose (#D16-10; Fisher Scientific) as carbon source. Cells were cultured for 4 h at 30°C with rotational shaking at 200 rpm in Erlenmeyer flasks at a “flask volume/medium volume” ratio of 5:1. A sample of cells was taken from a culture at a certain time-point and examined microscopically for the percentage of cells with new buds. At least 500 cells were examined per time point, and the budding percentage was calculated as follows: (number of cells with new buds per ml/total number of cells per ml) × 100%.

### Chronological lifespan measurement

A sample of cells was taken from a culture at a certain time point. A sample fraction was diluted to determine the total number of cells using a hemacytometer (#0267110; Fisher Scientific). Another fraction of the cell sample was diluted, and serial dilutions of cells were plated in duplicate onto YP plates containing 2% glucose as a carbon source. After 2 days of incubation at 30°C, the number of CFU per plate was counted. The number of CFU was defined as the number of viable cells in a sample. For each culture, the percentage of viable cells was calculated as follows: (number of viable cells per ml/total number of cells per ml) × 100. The percentage of viable cells in the mid-logarithmic phase was set at 100%.

### Measurement of glycogen and trehalose concentrations

2 × 10^9^ cells were harvested by centrifugation for 1 min at 16,000 × g at 4°C. The cell pellet was washed three times in ice-cold PBS (20 mM KH_2_PO_4_/KOH (pH 7.5) and 150 mM NaCl) and then resuspended in 200 μl of ice-cold SHE solution (50 mM NaOH and 1 mM EDTA [#E9884; Sigma-Aldrich]). 800 μl of ice-cold SHE solution were added to the cell suspension. The resulting alkali extract was incubated at 60°C for 30 min to destroy endogenous enzyme activities and pyridine nucleotides. The extract was neutralized by adding 500 μl of THA solution (100 mM Tris/HCl (pH 8.1) and 50 mM HCl). The extract was then divided into 150 μl aliquots, quickly frozen in liquid nitrogen and stored at –80°C before use.

To measure glycogen concentration, 50 μl of alkali extract were added to 500 μl of glycogen reagent (50 mM sodium acetate (pH 4.6) and 0.02% bovine serum albumin (BSA) [#A9418; Sigma-Aldrich] with and without of 10 μg/ml amyloglucosidase 6 U/mg [#11202367001; Sigma-Aldrich]). The mixture was incubated for 30 min at 25°C. 500 μl of glucose reagent (100 mM Tris/HCl (pH 8.1), 2 mM MgCl_2_, 1 mM 1,4-Dithiothreitol (DTT) [#D9163; Sigma-Aldrich], 1 mM ATP [#A7699; Sigma-Aldrich], 0.2 mM NADP^+^ [#10128040001; Sigma-Aldrich], and mixture of hexokinase (7 U; [11426362001; Sigma-Aldrich]) and glucose-6-phosphate dehydrogenase (8 U; [#G6378; Sigma-Aldrich]) were added, and the mixture was incubated for 30 min at 25°C. The NADPH generated from NADP^+^ was measured fluorimetrically (excitation at 365 nm, emission monitored at 460 nm).

To measure trehalose concentration, 50 μl of alkali extract were added to 150 μl of trehalose reagent (25 mM KH_2_PO_4_/KOH (pH 7.5) and 0.02% BSA [#A9418; Sigma-Aldrich] with or without 15 mU trehalase [#T8778; Sigma-Aldrich]). The mixture was incubated for 60 min at 37°C. 800 μl of glucose reagent (100 mM Tris/HCl (pH 8.1), 2 mM MgCl_2_, 1 mM DTT [#D9163; Sigma-Aldrich], 1 mM ATP [#A7699; Sigma-Aldrich], 0.2 mM NADP^+^ [#10128040001; Sigma-Aldrich], and mixture of hexokinase (7 U; [11426362001; Sigma-Aldrich]) and glucose-6-phosphate dehydrogenase (8 U) [#G6378; Sigma-Aldrich]) were added, and the mixture was incubated for 30 min at 25°C. The NADPH generated from NADP^+^ was measured fluorimetrically (excitation at 365 nm, emission monitored at 460 nm).

### Statistical analysis

Statistical analysis was performed using Microsoft Excel’s (2010) Analysis ToolPak - VBA. All data are presented as mean ± SEM. The *p* values for comparing the means of two groups using an unpaired two-tailed *t* test were calculated with the help of the GraphPad Prism 7 statistics software.

## Abbreviations

BSA: bovine serum albumin
CFU: colony-forming units
CL: cardiolipin
CLS: chronological lifespan
CR: caloric restriction
D: diauxic growth phase
DIC: differential interference contrast
DTT: 1,4-Dithiothreitol
HD: high-density cells
L: logarithmic growth phase
LCA: lithocholic acid
LD: low-density cells
NQ: non-quiescent cells
PD: post-diauxic growth phase
Q: quiescent cells
RCD: regulated cell death
ROS: reactive oxygen species
ST: stationary growth phase
TAG: triacylglycerols
YP: 1% yeast extract + 2% peptone
WT: wild-type strain
ΔΨ_m_: mitochondrial membrane potential
